# Relationships of SIGLEC family-related lncRNAs with clinical prognosis and tumor immune microenvironment in ovarian cancer

**DOI:** 10.1038/s41598-024-57946-7

**Published:** 2024-03-31

**Authors:** Xin Jin, Ying Chen, Qing Hu

**Affiliations:** 1 Department of Gynaecology, Shengjing Hospital of China Medical University, Shenyang, China; 2 Department of Ultrasound, Xiaoshan Traditional Chinese Medical Hospital, Hangzhou, China

**Keywords:** Ovarian cancer, SIGLEC family-related lncRNAs, Prognosis, Nomogram, Tumor microenvironment, Cancer, Immunology

## Abstract

Long non-coding RNAs (lncRNAs) and Sialic acid-binding immunoglobulin-type lectin (SIGLEC) family members play an important role in proliferation, apoptosis, immune-cell activation and tumor development. However, the relationships of SIGLEC family-related lncRNAs with clinical prognosis and tumor immune microenvironment in ovarian cancer (OC) are still unclear. 426 SIGLEC family-related lncRNAs were obtained according to the screening criteria R > 0.4 and p < 0.05 using Pearson correlation analysis. A risk model contained AL133279.1, AL021878.2, AC078788.1, AC039056.2, AC008750.1 and AC007608.3 was conducted based on the univariate Cox regression analysis, a least absolute shrinkage and selection operator (LASSO) Cox regression and multivariate Cox regression analyses. OC patient were divided into high-and low-risk group based on the median riskscore. K–M curve and ROC curve revealed that risk model has an abuset prognostic potential for OC patients. Moreover, we successfully validated the prognostic value of the model in the internal datasets, external datasets and clinical sample dataset. Finally, we found that the riskscore was positively correlated with the vast majority of immune cell infiltration. In conclusion, our research identified that a novel SIGLEC family-related lncRNAs risk model to predict the prognosis of OC patients. SIGLEC family-related lncRNAs risk model also has a positive relationship with the tumor immune microenvironment of OC, which may provide a new direction for immunotherapy of OC.

Ovarian cancer (OC) is the first leading cause of gynecological cancer death due to poor prognosis and diagnosing in advanced stage, causing about 300,000 new cases and 180,000 deaths annually worldwide^1,2^. Complete resection plus platinum-based chemotherapy has become the the primary choice for initial treatment of OC^3^. However, the 5-year survival rate remains below 45% because of its high metastasis and recurrence rate^4^. Therefore, it is imperative to identify new prognostic biomarkers and treatment strategy for OC.

Sialic acid (SA)-binding immunoglobulin (Ig)-like lectins (Siglecs) have two or three unique domains (an extracellular domain, a transmembrane domain and/or have no an intracellular domain) belonging to a family of Ig-like lectins^5,6^. Siglecs can be divided into two families according to the sequence homologs in hunman: conservative classic Siglecs containing Siglec-1 (CD169), Siglec-2 (CD22), Siglec-4 (MAG) and Siglec-15 and Siglec-3 (CD33)-related Siglecs including Siglec-3, Siglec-5, Siglec-6, Siglec-7, Siglec-8, Siglec-9, Siglec-10, Siglec-11, Siglec-12, Siglec-14 and Siglec-16^7,8^. Numerous studies have shown that cancer cells can express high levels of sialic acid surface antigens, which interact with Siglecs on leukocytes to perform immunosuppression and promote cancer escape. Shao et al.^9^ reported that Siglec-7 can promote the immune escape of tumor cells by inhibiting the killing function of NK cells. Läubli et al.^10^ found that inhibition of Siglec-9 can enhance the activity of neutrophils against tumor cells, implicating that Siglec-9 might be used as an immune suppressor or potential targets for immunotherapy. As an immunosuppressive factor, Siglic-15 is highly expressed on the surface of tumor cells, which is mutually exclusive with the expression of PD-L1, suggesting that Siglic-15 may be an important immune escape mechanism in patients with PD-L1 expression deletion^11^.

Long non-coding RNAs (lncRNAs) are nucleotide transcripts with over 200 nt in length. Numerous evidence indicated that lncRNAs play an important role in several biological processes such as migration, cell division, apoptosis, differentiation, metabolism and tumorigenesis^12^. For example, Li et al.^13^ identified that LncRNA DIAPH2-AS1 was overexpressed in Neural invasion (NI) positive gastric cancer (GC) tissues and was substantially associated with poor prognosis of GC patients. In vitro and in vivo experiments have shown that DIAPH2-AS1 may stimulate the invasion, migration and NI potential of GC cells by regulating NSUN2-NTN1 pathway. Liu et al.^14^ found that RP11-620J15 was upregulated in hepatocellular carcinoma (HCC) and was strongly linked to the tumor size and worse outcome of HCC. They also discovered that RP11-620J15.3 can promote the tumor progression and glycolytic pathway through miR-326/GPI axis. In addition, several studies have shown that lncRNAs can be involved in tumor progression by directly or indirectly targeting SIGLEC family genes. For instance, LINC00973 was overexpressed in Siglec-15-positive clear-cell renal cell carcinoma (ccRCC) and was involved in immune evasion through miR-7109-Siglec-15 pathway^15^. TUG1 has increased level in HCC tissues and cells and can enhance immunosuppression in HCC cells by targeting the hsa-miR-582-5p/Siglec-15 axis^16^. However, the connection between SIGLEC family genes and lncRNA-dependent OC progression is still unclear. Thus, it is crucial for us to clarify the potential connection between SIGLEC family genes and associated lncRNAs in OC.

In this research, we developed a SIGLEC family-related lncRNAs risk model to predict the prognosis of OC patients based on the Cancer Genome Atlas (TCGA) dataset. K–M curve and ROC curve revealed that risk model has an abuset prognostic potential for OC patients. Moreover, we successfully validated the predictive potential of the model in the internal datasets (TCGA-train dataset and TCGA-test dataset), external datasets (GSE9891 and GSE26193 datasets) and 54 clinical samples dataset. Finally, we found that the riskscore and SIGLEC family-related lncRNAs was positively correlated with the vast majority of immune cell infiltration, which may help with immunotherapy recommendations for OC patients.

## Materials and methods

### Data acquisition

**Figure 1:**
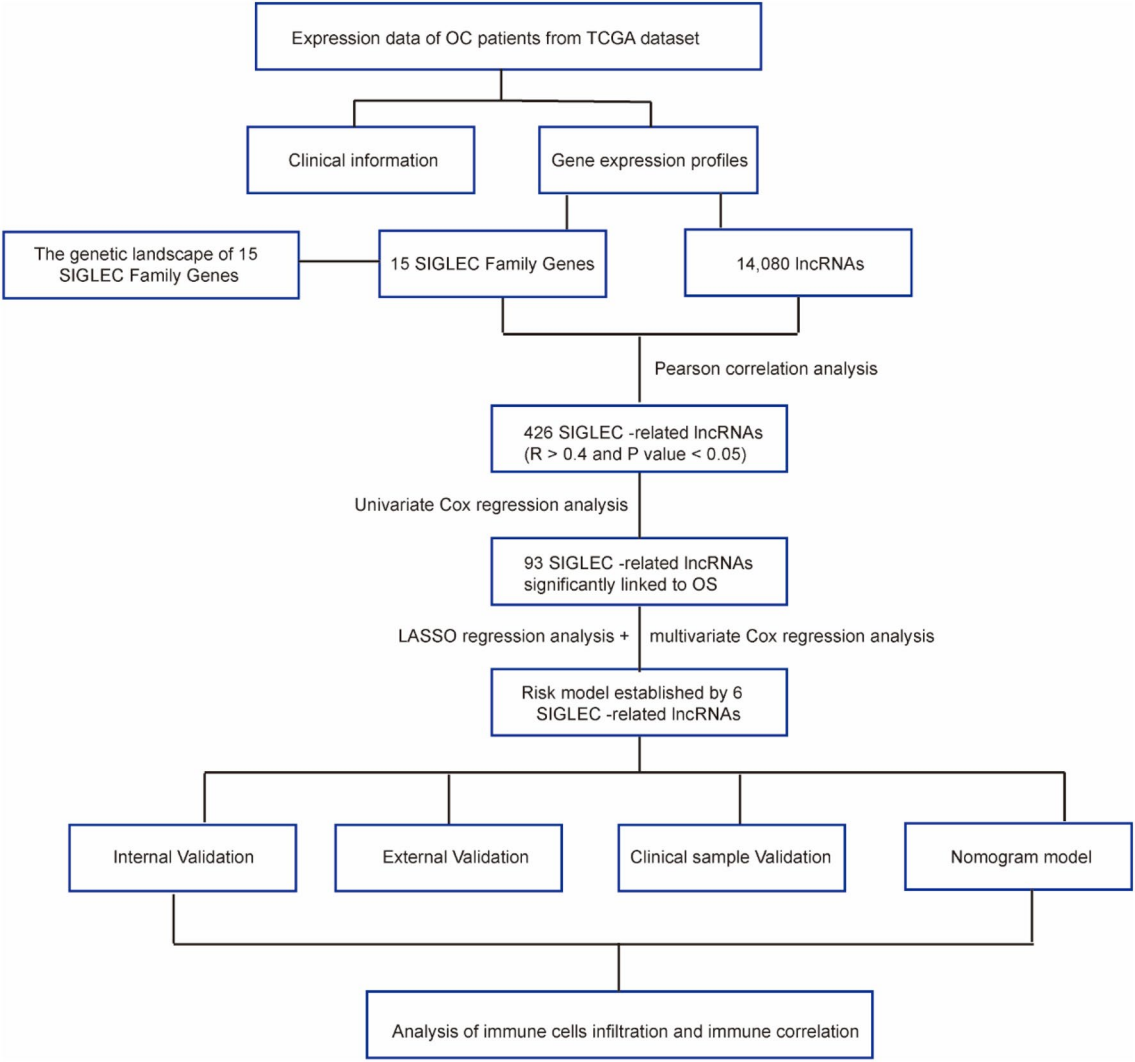
The overall workflow of the study. *TCGA* The Cancer Genome Atlas, *OC* ovarian cancer, *OS* overall survival.

The overall workflow of the study is presented in Fig. 1. The mRNAs-seq data, somatic mutation data, Copy Number Variation (CNV) data and clinical information of TCGA-OV dataset (379 OC samples) were downloaded from the Genomic Data Commons Data Portal (https://portal.gdc.cancer.gov/). The “oncoplot” was plotted using the “maftools”R package based on the somatic mutation data.The mRNAs-seq data and clinical information of GSE9891 (285 OC samples)^20^ and GSE26193 (107 OC samples)^21^ dataset were downloaded from Gene Expression Omnibus (GEO) database (https://www.ncbi.nlm.nih.gov/). We then normalized the raw data preprocessing of the two datasets was performed according to the standard procedure for Affymetrix microarray^17^. lncRNAs expression data were extracted from the mRNAs-seq data by Perl software.

### Specimen collection

A total of 54 OC samples were collected from Tissue specimen Bank of Shengjing Hospital from 2016 to 2022. None of the selected patients received any hormonal or chemoradiotherapy. This study was approved by the ethics committee of the ShengJing Hospital of China Medical University, and informed consent was obtained from all patients. In addition, all methods were executed in accordance with relevant guidelines and regulations.

### Identification of the SIGLEC family-related lncRNAs

The 15 SIGLEC family genes were extracted from the the mRNAs-seq data of TCGA-OV dataset. We calculated the correlation coefficient between 15 SIGLEC family genes and lncRNAs based on Pearson correlation analysis. Then, SIGLEC family-related lncRNAs were selected out acrroding to the screening criteria p < 0.05 and |R|> 0.4^23^.

### Construction and validation of the SIGLEC family-related lncRNAs prognostic signature

SIGLEC family-related lncRNAs with prognostic value were selected out based on the screening criteria p < 0.05 using univariate Cox regression analysis. Then, we further narrow the prognostic related SIGLEC family-related lncRNAs using A least absolute shrinkage and selection operator (LASSO) Cox regression and forward stepwise method, where the shrinkage and selection of variables were achieved through a logistic regression model with LASSO penalties, and λ-values were determined through iterative cross-validation^18^. Finally, we used multivariate Cox regression analysis to conduct a risk model and a riskscore for each OC patients were calculated according to the formula:

$$\begin{aligned} {\text{Risk score}} &= \sum {\text{Coef}}_{\text{i}}* \, {{\text{x}}_{\text{i}}}({\text{Coef}}_{\text{i}}\;{\text{represents the regression coefficient,}}\;{{\text{x}}_{\text{i}}}\;{\text{represents the expression }} \\ & \quad {\text{level of SIGLEC family-related lncRNAs}}). \\ \end{aligned}$$

OC patients were classified into high- and low-risk groups based on the median riskscore. Kaplan–Meier (K–M) method and The receiver-operating characteristics (ROC) curve was performed to evaluate the stability and suitability of the risk model. Univariate and multivariate Cox regression analyses was used to investigate whether the the riskscore is an independent prognostic factor for OC patients. The TCGA-OC patients was randomly divided into TCGA-train dataset (n = 188) and TCGA-test dataset (n = 187) and used as internal datasets. GSE9891 and GSE26193 were used as external datasets. Finally, we used the same methods mentioned above to validat our study results in internal datasets, external datasets and 54 clinical samples.

### Gene set variation analysis (GSVA) functional annotation

GSVA enrichment analysis was conducted using “GSVA” R package to explore the differential biological function between high- and low- risk groups based on the TCGA-OV, GSE9891 and GSE26193 datasets^19^. We downloaded the gene set c2.cp.kegg.v7.4.symbols.gmt from the MSigDB database (https://www.gsea-msigdb.org/gsea/msigdb). False discovery rate (FDR) was corrected by Benjamini and Hochberg (BH) method and FDR < 0.05 was considered as signatures.

### Real-time qPCR

We extracted the total RNA of 54 OC clinical samples using TriZol Reagent (Takara, Japan). the AMV reverse transcriptase reagent box (Takara, Japan) was used to carry out the cDNA synthesis. A 2 × SYBR Green PCR Master Mix was used to perform Real-time PCR. Next, GAPDH was served as an internal reference and the 2-ΔΔCt method was used to calculate the relative gene expression of the lncRNAs in risk model. The sequences of primers of the lncRNAs are presented in Supplementary Table 1.

### Construction of the nomogram model

A novel SIGLEC family-related lncRNAs nomogram, including the level of risk score and another three clinical factors (Age, Stage, and Grade), was constructed using the TCGA-OV dataset with the “regplot” and “rms” R packages. Calibration curves, Decision Curve Analysis (DCA) curve and ROC curve were drawn to assess the accuracy and clinical significance for prognostic prediction of OC patients^25^.

### The association of immunological characteristics and the prognostic risk model

Single sample gene set enrichment analysis (ssGSEA) algorithm were used to quantify the relative abundance of tumor immune-infiltrating cells (TIICs) in OC samples based on the TCGA-OV, GSE9891 and GSE26193 datasets^21^. Pearson correlation analysis was performed to investigate the correlation between immunological characteristics and genes and the prognostic risk model.

### Ethics approval and consent to participate

This study was approved by the ethics committee of the ShengJing Hospital of China Medical University, and informed consent was obtained from all patients. In addition, all methods were executed in accordance with relevant guidelines and regulations (2022PS401K).

## Results

### Landscape of SIGLEC family genes in TCGA-OV dataset

**Figure 2:**
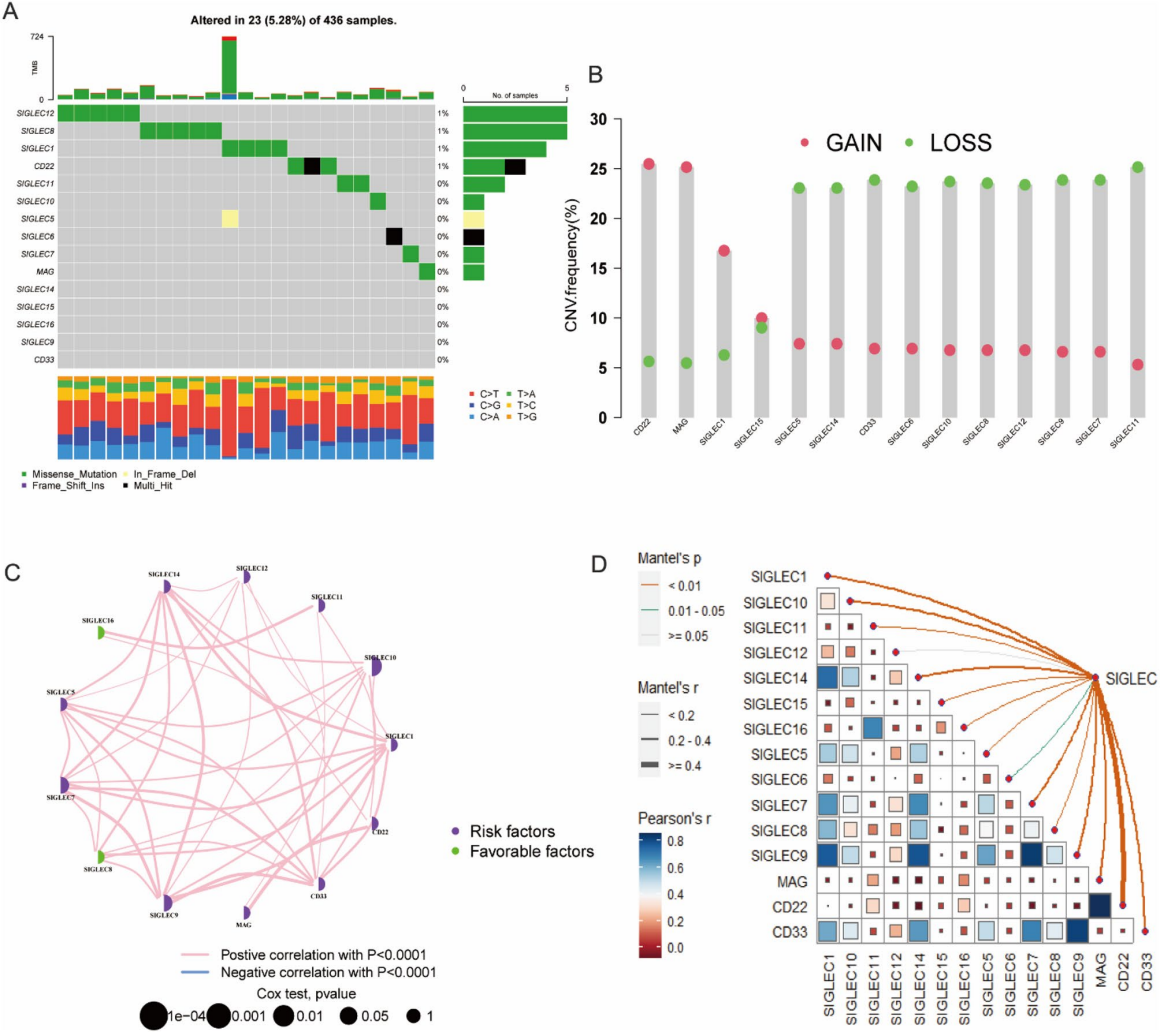
Genetic and transcriptional alterations of SIGLEC family genes in OC. (**A**) Mutation frequencies of the 15 SIGLEC family genes. (**B**) Frequencies of CNV gain, loss, and non-CNV among the 15 SIGLEC family genes. (**C**) The interactions between 15 SIGLEC family genes. (**D**) The correlations among the 15 SIGLEC family genes. *TCGA* The Cancer Genome Atlas, *OC* ovarian cancer, *CNV* copy number variant.

**Figure 3:**
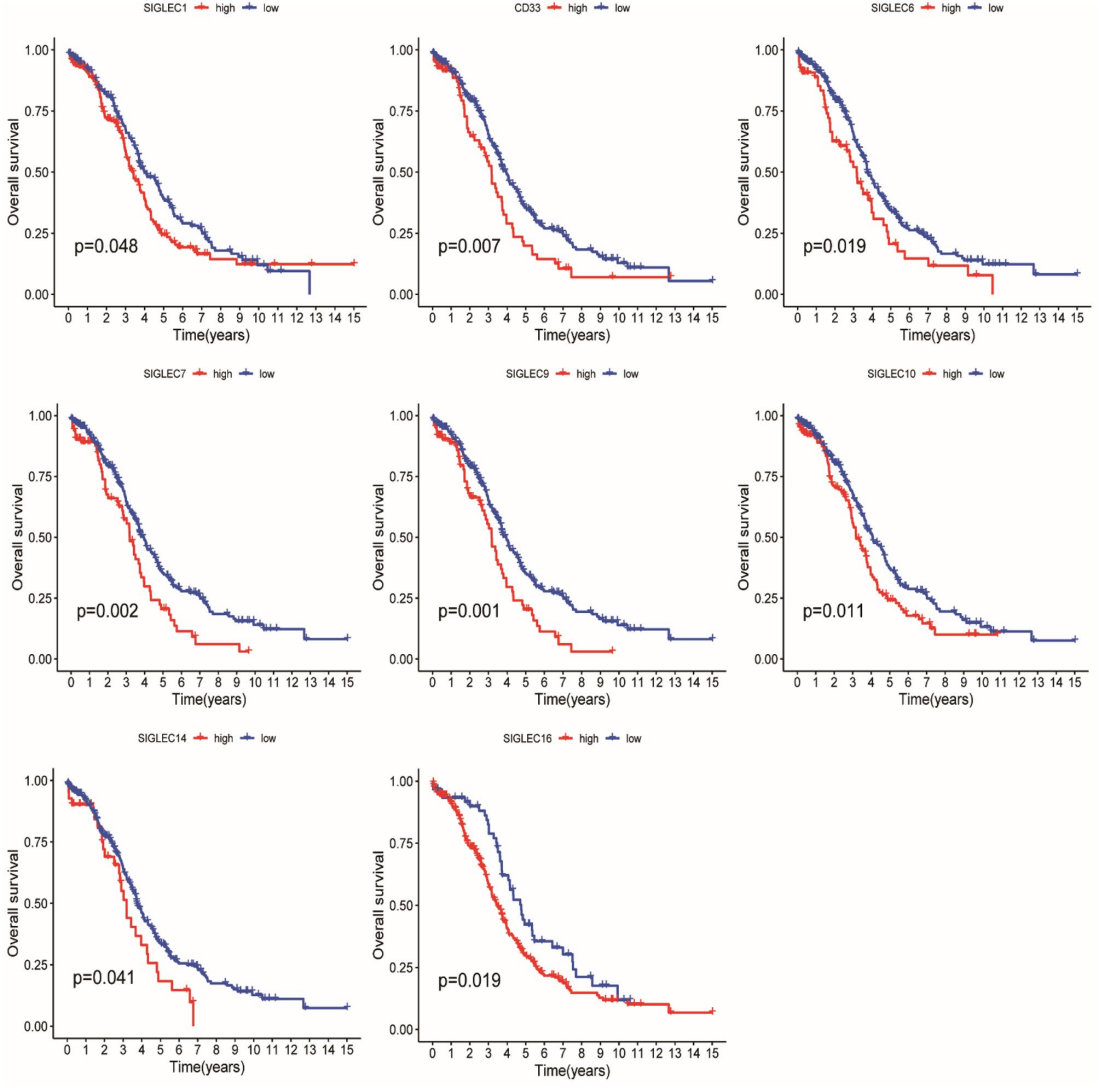
The 15 SIGLEC family genes with significant prognostic value in OC.

In the TCGA-ov dataset, the somatic mutation frequency of 15 SIGLEC family genes were not very frequent. Of the 436 OC samples, 23 (5.28%) had mutations of SIGLEC family genes (Fig. 2A). CNV alteration frequency showed that conservative classic Siglecs had higher frequency of CNV gain than CNV loss, while Siglec-3 (CD33)-related Siglecs had a higher proportion of CNV loss than CNV gain (Fig. 2B). The interactions, correlation and prognostic value of the 15 SIGLEC family genes in TCGA-OV dataset were presented in Figs. 2C,D, 3. We found that the majority of the 15 SIGLEC family genes have positive correlation with each other. The expression level of Siglec-1, CD33, Siglec-6, Siglec-7, Siglec-9, Siglec-10, Siglec-14 and Siglec-16 were asscociated with poor outcome of OC patients (Fig. 3).

### Construction and validation of the SIGLEC family-related lncRNAs prognostic signature

**Figure 4:**
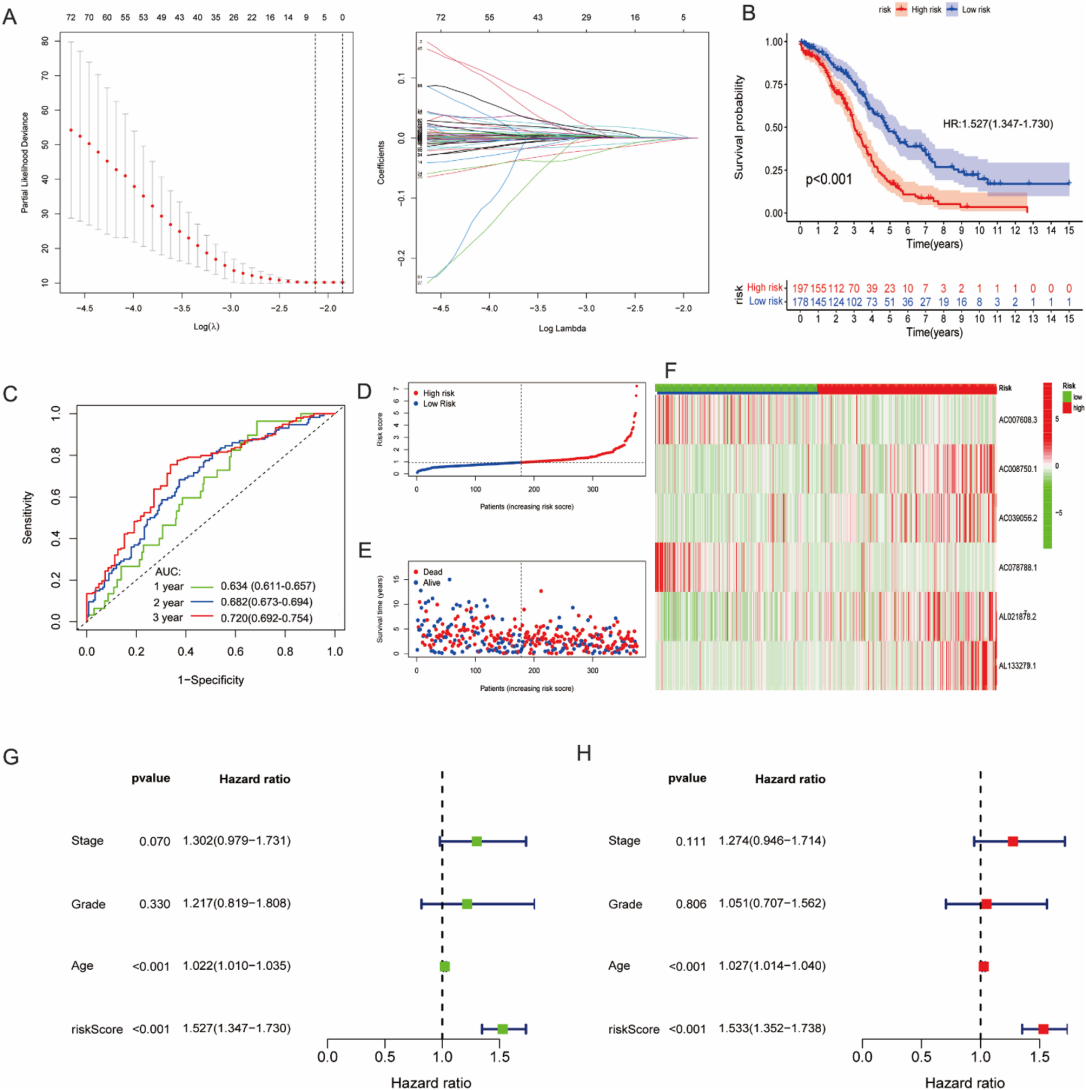
Identification of six SIGLEC family-related lncRNAs prognostic signature. (**A**) LASSO regression was applied to establish the SIGLEC family-related lncRNAs prognostic signature. (**B**) K–M survival curve showed survival analysis of the six SIGLEC family-related lncRNAs prognostic signature. (**C**) ROC curve analysis of the six SIGLEC family-related lncRNAs prognostic signature. (**D**–**F**) riskcore, survival status and heatmap of the six SIGLEC family-related lncRNAs. (**G**,**H**) Univariate and multivariate Cox analysis to assess the independent prognostic value of the six SIGLEC family-related lncRNAs prognostic signature. *LASSO* Least Absolute Shrinkage and Selection Operator, *K–M* Kaplan–Meier, *ROC* receiver operating characteristic.

426 SIGLEC family-related lncRNAs were obtained using pearson correlation analysis (Supplementary Table 2). Then, we selected out 93 SIGLEC family-related lncRNAs with prognostic value using univariate Cox regression analysis (Supplementary Table 3). Lasso Cox regression analysis was performed to narrow down the number of SIGLEC family-related lncRNAs, with 7 lncRNAs left as lambda = 0.0332 (Fig. 4A). Finally, we selected out 6 SIGLEC family-related lncRNAs in the risk signature after multivariate Cox regression analysis with stepwise regression method forward stepwise regression. A riskscore for each OC patients were calculated according to the formula: riskscore = (− 0.01511 * AC007608.3) + (0.004533 * AC008750.1) + (0.004272 * AC039056.2) + (− 0.02664 * AC078788.1) + (0.00455 * AL021878.2) + (0.030881 * AL133279.1) (Supplementary Table 4). Patients were classified into high- and low- risk groups based on the the median score as the threshold. The prognostic value of this signature OC patients was validated by performing K–M and time-dependent ROC analyses. The results showed that patients with low risk had an extremely better overall survival (OS) rate (Fig. 4B). Area under the curve (AUC) values for predicting 1-, 3-, and 5-year OS were 0.634 (95% CI 0.611–0.657), 0.682 (95% CI 0.673–0.694)and 0.770 (95% CI 0.692–0.754), respectively (Fig. 4C). Figure 4D–F represent riskcore, survival status and heatmap of six SIGLEC family-related lncRNAs, respectively. Univariate (HR = 1.527, 95% CI 1.347–1.730, p < 0.001)and multivariate Cox regression analyses (HR = 1.533, 95% CI 1.352–1.738, p < 0.001) indicated that riskscore remained independent prognostic indicators of unfavorable OS (Fig. 4G,H). In addition, we plotted heatmap to reveal the positive correction between six SIGLEC family-related lncRNAs and 15 SIGLEC family genes (Supplementary Fig. 1A). Supplementary Fig. 1B have shown that the riskscore correlated strongly positively with the expression level of CD33, Siglec-1, Siglec-5, Siglec-7, Siglec-9, Siglec-10 and Siglec-14.

**Figure 5:**
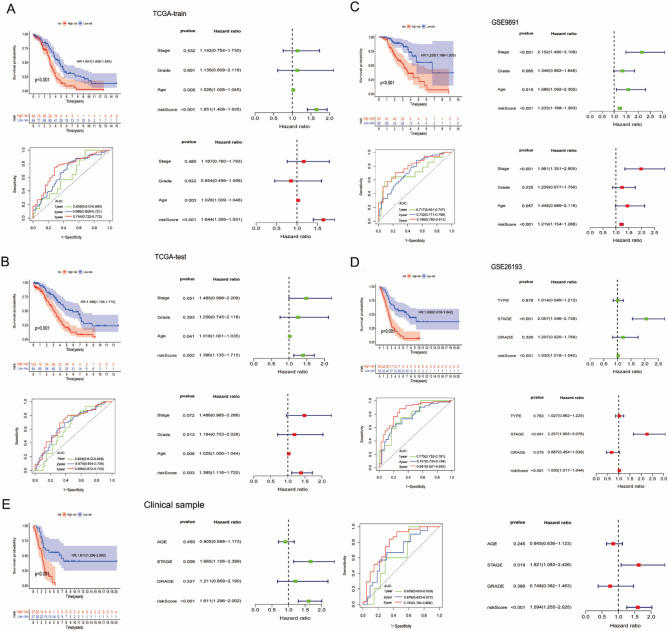
Validation of the six SIGLEC family-related lncRNAs prognostic signature. (**A**) Verify in the TCGA-train dataset. (**B**) Verify in the TCGA-test dataset. (**C**) Verify in the GSE9891 dataset. (**D**) Verify in the GSE26193 dataset. (**E**) Verify in the clinical specimens.

To confirm the prognostic value of our SIGLEC family-related lncRNAs prognostic signature identified in the TCGA-OV dataset, internal datasets, external datasets and 54 clinical samples were used to test our results. With the same calculation formula and median value of the generated risk scores, the OC patients were correspondingly segregated into low-risk and high-risk groups. In survival analysis, patients in the low-risk group showed longer OS than those in the high-risk group in the datasets TCGA- train (HR = 1.651, 95% CI 1.408–1.935, p < 0.001), TCGA- test (HR = 1.396, 95% CI 1.135–1.715, p < 0.001), GSE9891 (HR = 1.233, 95% CI 1.166–1.303, p < 0.001), GSE26193 (HR = 1.030, 95% CI 1.018–1.042, p < 0.001) and clinical samples (HR = 1.611, 95% CI 1.296–2.002, p < 0.001), which was consistent with the results (Fig. 5). Area under the curve (AUC) values for predicting 1-, 3-, and 5-year OS in the datasets TCGA- train, TCGA- test, GSE9891, GSE26193 and clinical samples were all greater than 0.6, suggesting that the predictive ability of the prognosis prediction model was stable (Fig. 5).

**Figure 6:**
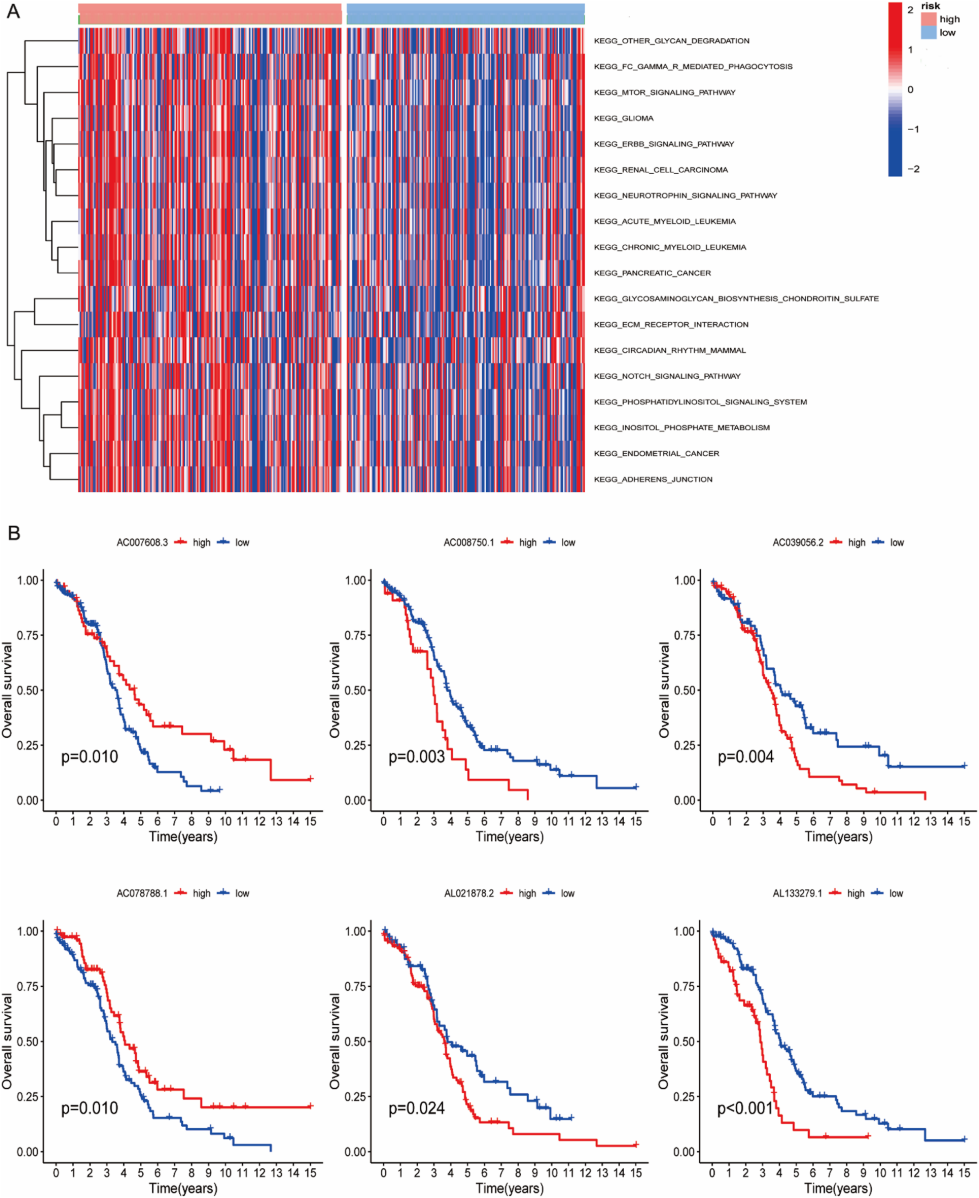
GSVA functional annotation. (**A**) The differences in KEGG pathways between high- and low-risk group. (**B**) The prognostic value of six SIGLEC family-related lncRNAs in OC patients.

Given that the prognostic value of the SIGLEC family-related lncRNAs prognostic signature was fully assessed, we attempted to explore the underlying mechanism. GSVA enrichment analysis between high- and low- risk groups revealed that KEGG pathways related to MTOR SIGNALING PATHWAY, ERBB SIGNALING PATHWAY and NOTCH SIGNALING PATHWAY were enriched in high riskscore group (Fig. 6A). In addition, K–M curve indicated that all of the six lncRNAs in the SIGLEC family-related lncRNAs prognostic signature were asscociated with worse prognosis of OC patients (Fig. 6B).

### Construction of the nomogram model

**Figure 7:**
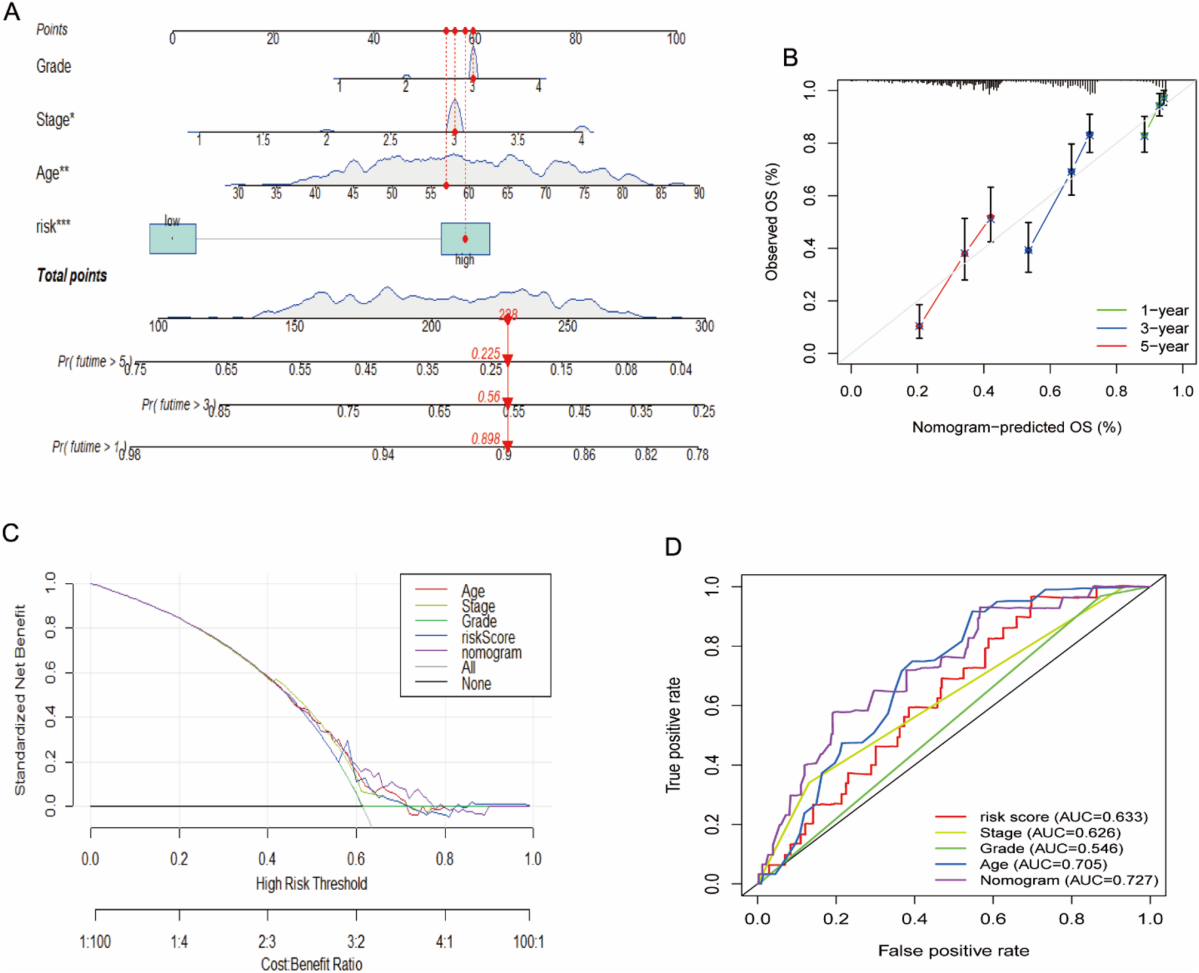
Construction of the nomogram model based on TCGA-OV dataset. (**A**) A nomogram for predicting the 1-, 3-, 5-year overall survival rates of OC patients. (**B**) The calibration curve at 1-, 3-, 5-year. (**C**) DCA curves for predicting the OS of different parameters. (**D**) ROC curves for predicting the OS of different parameters. *OC* ovarian cancer, *DCA* clinical decision curve, *ROC* receiver operating characteristic, *OS* overall survival.

Based on these riskscore and clinical information, we developed a prognostic nomogram that incorporates risk score stratification and three other clinicopathologic factors, including age, stage and grade, for predicting individual OS at 1, 3, and 5 years (Fig. 7A), and calibration plots demonstrated the stable performance of the nomogram (Fig. 7B). DCA curve suggested that our nomogram showed higher net benefit when compared with the other single factor (Fig. 7C). Moreover, the nomogram showed increased predictive accuracy when compared with the individual feature (Fig. 7D). Together, the predictive performance of the nomogram based on the risk score and clinical information is useful for survival prediction of OC patients.

### The association of immunological characteristics and the prognostic risk model

**Figure 8:**
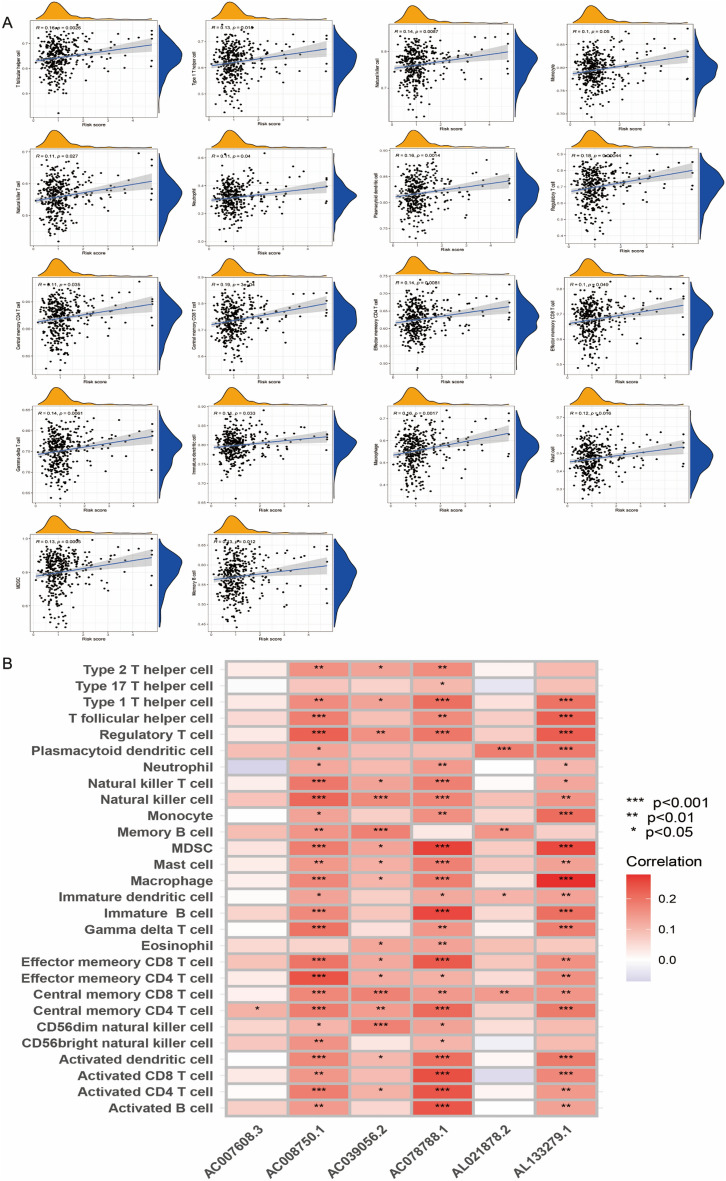
The association of immunological characteristics and the prognostic risk model. (**A**) The correlation between the SIGLEC family-related lncRNAs prognostic signature and TIICs. (**B**) The correlation between the six SIGLEC family-related lncRNAs and the abundance of TIICs.

Considering the close correlation between the SIGLEC family-related lncRNAs prognostic signature and activities of the immune response, we evaluated infiltration levels of diverse TIICs between the two risk groups to reveal immune microenvironment landscapes. According to our results, the vast majority of immune cells were more likely to infiltrate tumors of the high-risk group (Fig. 8A). Correlation analysis was then performed between the abundance of TIICs and the SIGLEC family-related lncRNAs, and the AC008750.1, AC039056.2, AC078788.1 and AL133279.1 were correlated strongly positively with the TIICs (Fig. 8B). Collectively, these results comprehensively reveal distinct immune features between high- and low-risk and OC patients bearing high-risk tumors are more likely to benefit from immune checkpoint inhibitors.

## Discussion

In this reaearch, we extracted 14,080 lncRNAs and 15 SIGLEC family genes expression data from TCGA-OV dataset to identify SIGLEC family-related lncRNAs prognostic significance in OC. Then, 93 SIGLEC family-related lncRNAs with prognostic value were selected out using univariate Cox regression analysis acrroding to the screening criteria p < 0.05 and |R|> 0.4. Next, six SIGLEC family-related lncRNAs selected by LASSO Cox regression were used to conduct a SIGLEC family-related lncRNAs prognostic signature. Patients in TCGA-OV dataset were divided into high- and low- risk groups acrroding to the the median score, and the patients in low- risk group have longer survival time. We also successfully verified our results in internal and external datasets. Finally, we evaluated infiltration levels of diverse TIICs between the two risk groups to reveal immune microenvironment landscapes and found that the vast majority of immune cells were more likely to infiltrate tumors of the high-risk group, indicating that OC patients bearing high-risk tumors are more likely to benefit from immune checkpoint inhibitors.

Increased studies have shown that SIGLEC family genes were strongly related to the development of cancers. Zhou et al.^20^ indicated that Siglec-15 expression was positivity related to a more favorable pathological response and better prognosis of esophageal squamous cell carcinoma patients with neoadjuvant chemoradiotherapy. Benmerzoug et al.^21^ reported that levels of Siglic-7 protein in blood, urine, and tumor tissue of bladder cancer (BCa) patients were involved in poor survival and NK cell-mediated antitumor immunity. Fan et al.^22^ have shown that upregulated Siglec-15 was associated with the poor clinical outcomes of Osteosarcoma (OS) patients and promoted the proliferation, migration and invasion of OS cells by regulating DUSP1/MAPK Pathway. However, he connection between SIGLEC family genes and lncRNA-dependent OC progression remains unclear. In our study, we obtained the potential interaction between the SIGLEC family genes and lncRNAs, but more fundamental research in vivo and in vitro is still needed to be done in the future.

A six SIGLEC family-related lncRNAs prognostic signature (AC007608.3, AC008750.1, AC039056.2, AC078788.1, AL021878.2, AL133279.1) was established to predict the OS of OC patients and all of them have close correction with worse prognosis of OC. Previous research has shown that AC007608.3 was related to cuproptosis and poor prognosis of colorectal cancer^23^. Feng et al.^24^ revealed that AC078788.1 was associated with tumor immunity and a favorable prognosis of OC. AC008750.1 has been reported to be related to the malignant progression of BCa^25^, OC^26^, lung adenocarcinoma (LUAD)^27^ and oral squamous cell carcinoma (OSCC)^28^. However, other three SIGLEC family-related lncRNAs have not been reported yet. GSVA enrichment analysis was performed to explore the underlying mechanism between high- and low- risk groups. The results revealed that KEGG pathways related to MTOR SIGNALING PATHWAY, ERBB SIGNALING PATHWAY and NOTCH SIGNALING PATHWAY were more enriched in high-risk group and may contribute to the worse outcome.

This study had certain limitations. Firstly, since the sample tissues are from individual tissues rather than blood,we could not determine whether the six SIGLEC family-related lncRNAs in the risk model can be tested in a blood sample. Secondly, we need further experiment to verify our results and carry on in-depth mechanism studies due to the majority of results in our research come from bioinformatics analysis. In the future, we will collect a certain number of fresh OC samples to make paraffin sections, and use immunohistochemistry staining to verify the correlation between the SIGLEC family-related lncRNAs prognostic signature and activities of the immune cell. In addition, our research also has some advantages. Firstly, we evaluated the predictive value, immunotherapy efficacy of the prognostic model for OC patients, providing a basis for the immunotherapy for OC patients. Secondly, we incorporated omics information from several dimensions of OC to fully leverage the informative content of each omics dimension.

## Conclusions

Overall, our research succeeded in conducting a six SIGLEC family-related lncRNAs prognostic signature to predict the OS of OC patients. More importantly, the six SIGLEC family-related lncRNAs are closely related to the tumor immune microenvironment of OC, providing a theoretical foundation for immunotherapy of OC patients.

### Supplementary Information

Supplementary Information 1.

Supplementary Information 2.

Supplementary Information 3.

Supplementary Information 4.

Supplementary Information 5.

## Data Availability

The datasets generated and/or analysed during the current study are available in the TCGA (https://portal.gdc.cancer.gov/) and GEO (https://www.ncbi.nlm.nih.gov/) repository.

## Abbreviations

lncRNAs: Long non-coding RNAs
OC: Ovarian cancer
TCGA: The Cancer Genome Atlas
GEO: Gene Expression Omnibus
LASSO: Least absolute shrinkage and selection operator
K–M: Kaplan–Meier
ROC: Receiver-operating characteristics
AUC: Area under the curve
OS: Overall Survival
