# Supplementary material for: Relationships of SIGLEC family-related lncRNAs with clinical prognosis and tumor immune microenvironment in ovarian cancer
Source: Sci Rep. 2024 Mar 31;14:7593. doi: 10.1038/s41598-024-57946-7 (PMC10982283; doi:10.1038/s41598-024-57946-7)
Supplement: Supplementary file 1 — Supplementary Information 1. [file 41598_2024_57946_MOESM1_ESM.docx]

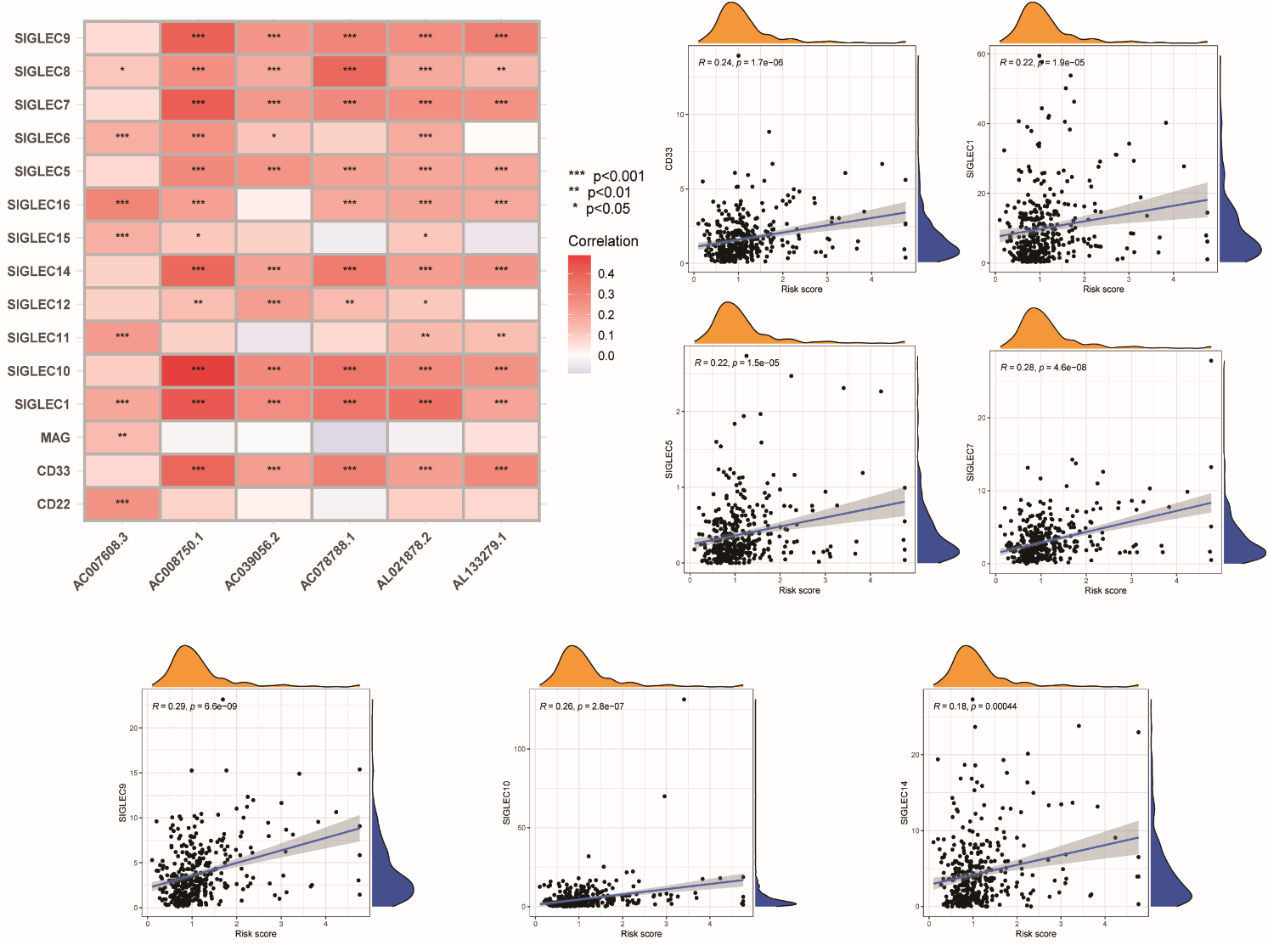


**Supplementary figure 1.** The correction between six SIGLEC family-related lncRNAs and 15 SIGLEC family genes.
