# Supplementary material for: Relationships of SIGLEC family-related lncRNAs with clinical prognosis and tumor immune microenvironment in ovarian cancer
Source: Sci Rep. 2024 Mar 31;14:7593. doi: 10.1038/s41598-024-57946-7 (PMC10982283; doi:10.1038/s41598-024-57946-7)
Supplement: Supplementary file 2 — Supplementary Information 2. [file 41598_2024_57946_MOESM2_ESM.docx]

Supplementary table 1: Primer sequences of the six lncRNAs for reverse transcription- quantitative polymerase chain reaction.

| **Gene** | **Forward (5′-3′)** | Reverse (5′-3′) |
| --- | --- | --- |
| AL133279.1 | 5'-CATGTATGGAGGCTGACCTGTATGC-3' | 5'-CGTGGTTCATAGTAGCTGGGTTGG-3' |
| AL021878.2 | 5'-CCGTGTTAGCCAGAATGGTCTCG-3' | 5'-GCCCAAGGTCACACAGCTAGTTG-3' |
| AC078788.1 | 5'-GGACTTGATGACTGCGGTGTGG-3' | 5'-TGTGCTGAGTGGTTCAATCGACTG-3' |
| AC039056.2 | 5'-TGCCTTGTGGGACTACAGACCTG-3' | 5'-TGCTGGCTTCAATGCTGTCACTC-3' |
| AC008750.1 | 5'-GGAATCGGCATCACGGCTCTTC-3' | 5'-CTGGGAAACTGGGCAACTGATACTG-3' |
| AC007608.3 | 5'-GGGACAGCCACTAGAGAGCAGAG-3' | 5'-AGGGACAGCACCAGAACCAGAC-3' |
| GAPDH | 5'-TGACAACTTTGGTATCGTGGAAGG-3' | 5'-AGGCAGGGATGATGTTCTGGAGAG-3' |
