# Supplementary material for: Relationships of SIGLEC family-related lncRNAs with clinical prognosis and tumor immune microenvironment in ovarian cancer
Source: Sci Rep. 2024 Mar 31;14:7593. doi: 10.1038/s41598-024-57946-7 (PMC10982283; doi:10.1038/s41598-024-57946-7)
Supplement: Supplementary file 3 — Supplementary Information 3. [file 41598_2024_57946_MOESM3_ESM.docx]

Supplementary table 2. Four hundred and twenty-six SIGLEC family -related lncRNAs was obtained through pearson correlation analysis in TCGA-OV dataset.

| GENE1 | GENE2 | P | R |
| --- | --- | --- | --- |
| LINC00924 | SIGLEC11 | 4.41E-126 | 0.884785 |
| AC126773.4 | SIGLEC11 | 1.39E-114 | 0.865935 |
| AC024337.2 | SIGLEC11 | 3.60E-114 | 0.865198 |
| AC092155.1 | SIGLEC11 | 5.45E-110 | 0.857497 |
| LINC01197 | SIGLEC11 | 1.23E-107 | 0.85295 |
| AC010636.1 | SIGLEC11 | 1.52E-107 | 0.852772 |
| U62631.1 | CD22 | 2.65E-105 | 0.848289 |
| AL355612.1 | SIGLEC11 | 4.57E-104 | 0.84575 |
| AC011899.2 | SIGLEC9 | 3.46E-103 | 0.843919 |
| AL353611.1 | SIGLEC11 | 2.81E-97 | 0.830962 |
| AC022166.1 | SIGLEC11 | 3.07E-97 | 0.830876 |
| AC090559.1 | SIGLEC9 | 7.99E-95 | 0.825237 |
| MEF2C-AS1 | SIGLEC11 | 1.16E-93 | 0.822451 |
| LINC01262 | SIGLEC11 | 1.30E-93 | 0.822332 |
| U62631.1 | MAG | 2.91E-93 | 0.821481 |
| GRTP1-AS1 | SIGLEC11 | 1.32E-92 | 0.819874 |
| LINC01094 | SIGLEC9 | 6.98E-89 | 0.810456 |
| LINC01391 | SIGLEC11 | 1.50E-84 | 0.798787 |
| AC010776.2 | SIGLEC11 | 1.27E-81 | 0.790447 |
| LINC02360 | SIGLEC11 | 5.73E-77 | 0.776341 |
| AC011899.2 | CD33 | 3.11E-76 | 0.774017 |
| AP003774.2 | SIGLEC10 | 6.50E-75 | 0.76977 |
| NR2F2-AS1 | SIGLEC11 | 2.33E-73 | 0.764645 |
| AC090559.1 | CD33 | 5.30E-71 | 0.756623 |
| AC145098.1 | SIGLEC9 | 1.30E-69 | 0.751741 |
| MIR202HG | SIGLEC11 | 8.43E-69 | 0.748838 |
| C8orf49 | SIGLEC11 | 2.20E-66 | 0.739945 |
| LINC02126 | SIGLEC11 | 4.61E-66 | 0.738736 |
| LINC01150 | SIGLEC9 | 5.73E-66 | 0.738378 |
| AC138207.1 | SIGLEC1 | 7.12E-66 | 0.73802 |
| CCR5AS | SIGLEC9 | 7.61E-64 | 0.730192 |
| AC023154.1 | SIGLEC11 | 1.85E-63 | 0.72867 |
| LINC01094 | CD33 | 2.66E-63 | 0.728046 |
| AC020913.1 | SIGLEC12 | 7.29E-63 | 0.726306 |
| LINC01543 | SIGLEC10 | 1.52E-62 | 0.725027 |
| AC104260.1 | SIGLEC11 | 5.32E-62 | 0.722829 |
| AC110995.1 | SIGLEC1 | 1.79E-61 | 0.720679 |
| AC011899.2 | SIGLEC7 | 2.33E-60 | 0.716071 |
| AC110995.1 | SIGLEC9 | 9.74E-60 | 0.713461 |
| AL021026.1 | SIGLEC12 | 1.58E-59 | 0.712568 |
| AC012409.3 | SIGLEC11 | 8.21E-59 | 0.709516 |
| OVCH1-AS1 | SIGLEC12 | 3.78E-58 | 0.70665 |
| LINC02285 | SIGLEC9 | 7.80E-58 | 0.705276 |
| AC145098.1 | SIGLEC1 | 4.06E-57 | 0.702122 |
| LINC01150 | CD33 | 9.99E-57 | 0.700379 |
| LINC02683 | SIGLEC12 | 4.08E-56 | 0.697633 |
| AL583803.1 | SIGLEC11 | 3.12E-55 | 0.693611 |
| AC120498.10 | SIGLEC11 | 6.85E-54 | 0.687371 |
| ADAMTS9-AS2 | SIGLEC11 | 8.21E-54 | 0.687001 |
| AC011899.2 | SIGLEC14 | 1.05E-53 | 0.68649 |
| HLCS-IT1 | SIGLEC12 | 2.13E-53 | 0.685034 |
| CCR5AS | SIGLEC14 | 3.08E-53 | 0.684277 |
| AC068944.1 | SIGLEC11 | 8.87E-53 | 0.682074 |
| AC006033.2 | SIGLEC9 | 1.71E-52 | 0.680702 |
| LINC01094 | SIGLEC14 | 1.93E-52 | 0.680447 |
| CCR5AS | CD33 | 2.38E-52 | 0.680003 |
| AC090559.1 | SIGLEC14 | 4.50E-52 | 0.678655 |
| AC090559.1 | SIGLEC7 | 7.31E-52 | 0.677627 |
| AC110995.1 | SIGLEC14 | 1.05E-51 | 0.676852 |
| LINC01094 | SIGLEC7 | 1.47E-51 | 0.676132 |
| LINC02285 | CD33 | 2.23E-51 | 0.675241 |
| AC090559.1 | SIGLEC1 | 2.61E-51 | 0.674904 |
| AL355607.2 | SIGLEC12 | 2.69E-51 | 0.67484 |
| AC060234.1 | SIGLEC11 | 3.14E-51 | 0.674508 |
| AL590764.1 | SIGLEC9 | 3.55E-50 | 0.669228 |
| AC091151.1 | SIGLEC15 | 5.46E-50 | 0.66828 |
| MRGPRF-AS1 | SIGLEC11 | 6.10E-50 | 0.668036 |
| AC005999.1 | SIGLEC11 | 8.78E-50 | 0.667231 |
| AC011899.2 | SIGLEC1 | 2.46E-49 | 0.664939 |
| AL049629.1 | SIGLEC12 | 3.22E-49 | 0.664332 |
| AC006033.2 | SIGLEC14 | 5.33E-49 | 0.663205 |
| LINC00989 | SIGLEC11 | 1.37E-47 | 0.655784 |
| AC002128.1 | MAG | 1.69E-47 | 0.655306 |
| AL357146.1 | SIGLEC11 | 1.92E-46 | 0.649605 |
| AC011726.3 | SIGLEC11 | 2.37E-46 | 0.649106 |
| AC016229.1 | SIGLEC11 | 7.05E-46 | 0.646503 |
| AL035425.2 | SIGLEC11 | 7.61E-46 | 0.646322 |
| AC145098.1 | CD33 | 9.43E-46 | 0.645806 |
| AC025280.2 | SIGLEC11 | 4.92E-45 | 0.641803 |
| AC138207.1 | SIGLEC9 | 1.46E-44 | 0.639136 |
| AC100849.1 | SIGLEC1 | 2.37E-44 | 0.637934 |
| AC145098.1 | SIGLEC14 | 4.54E-44 | 0.636323 |
| AC145098.1 | SIGLEC7 | 8.94E-44 | 0.63463 |
| AP001043.1 | SIGLEC12 | 9.67E-44 | 0.634433 |
| AC087477.5 | SIGLEC11 | 1.17E-43 | 0.633966 |
| AL590764.1 | SIGLEC14 | 1.46E-43 | 0.6334 |
| LINC02643 | SIGLEC11 | 1.89E-43 | 0.632753 |
| AL590764.1 | CD33 | 1.89E-43 | 0.632751 |
| PRKAR1B-AS1 | SIGLEC9 | 6.84E-43 | 0.629482 |
| ZNF30-AS1 | MAG | 1.11E-42 | 0.628242 |
| TRG-AS1 | SIGLEC8 | 1.76E-42 | 0.627063 |
| AC138512.1 | SIGLEC11 | 5.58E-42 | 0.624069 |
| AC011700.1 | SIGLEC16 | 7.19E-42 | 0.623406 |
| AC006033.2 | SIGLEC1 | 9.61E-42 | 0.622648 |
| CCR5AS | SIGLEC7 | 1.21E-41 | 0.62204 |
| LINC01094 | SIGLEC1 | 1.75E-41 | 0.621081 |
| AC010776.3 | SIGLEC16 | 2.01E-41 | 0.620706 |
| AL354861.3 | SIGLEC11 | 3.36E-41 | 0.619351 |
| CARMN | SIGLEC11 | 4.88E-41 | 0.61836 |
| LINC01146 | SIGLEC14 | 5.45E-41 | 0.618065 |
| AC138207.5 | SIGLEC9 | 6.26E-41 | 0.617696 |
| ADAMTS9-AS1 | SIGLEC11 | 1.28E-40 | 0.615775 |
| AC110995.1 | SIGLEC7 | 2.24E-40 | 0.614282 |
| LINC01537 | SIGLEC11 | 3.41E-40 | 0.61314 |
| PCED1B-AS1 | SIGLEC9 | 3.63E-40 | 0.612969 |
| AC018647.1 | SIGLEC11 | 4.52E-40 | 0.612379 |
| AL355612.1 | SIGLEC16 | 1.08E-39 | 0.610006 |
| AC007728.2 | SIGLEC14 | 1.61E-39 | 0.608905 |
| AL139351.1 | SIGLEC11 | 1.80E-39 | 0.608601 |
| AC016229.1 | SIGLEC16 | 5.34E-39 | 0.605586 |
| AL133371.2 | SIGLEC9 | 5.86E-39 | 0.605327 |
| LINC01150 | SIGLEC14 | 7.34E-39 | 0.604698 |
| AC084064.1 | SIGLEC11 | 8.24E-39 | 0.604377 |
| SMIM25 | SIGLEC9 | 8.46E-39 | 0.604304 |
| LINC01146 | SIGLEC9 | 1.17E-38 | 0.603403 |
| AC110995.1 | CD33 | 1.34E-38 | 0.603019 |
| LINC01146 | SIGLEC8 | 1.39E-38 | 0.602919 |
| AC006065.4 | SIGLEC12 | 2.34E-38 | 0.60145 |
| AC006033.2 | SIGLEC8 | 2.85E-38 | 0.600882 |
| LINC02285 | SIGLEC7 | 5.47E-38 | 0.599037 |
| AC027601.3 | SIGLEC11 | 5.74E-38 | 0.598897 |
| AC245128.3 | SIGLEC9 | 8.31E-38 | 0.597842 |
| AC011899.2 | SIGLEC5 | 9.40E-38 | 0.597488 |
| AC006033.2 | CD33 | 1.21E-37 | 0.596754 |
| AL590764.1 | SIGLEC7 | 1.23E-37 | 0.596724 |
| AC100849.1 | SIGLEC9 | 1.87E-37 | 0.595519 |
| PIK3CD-AS1 | SIGLEC9 | 2.33E-37 | 0.594877 |
| PIK3CD-AS1 | SIGLEC1 | 3.85E-37 | 0.593423 |
| LINC00626 | SIGLEC16 | 3.98E-37 | 0.593323 |
| MROCKI | SIGLEC8 | 4.20E-37 | 0.593167 |
| AC023796.1 | MAG | 8.84E-37 | 0.590997 |
| LINC01985 | SIGLEC11 | 1.13E-36 | 0.590274 |
| AC099560.1 | SIGLEC12 | 1.16E-36 | 0.590212 |
| TTTY7 | MAG | 1.49E-36 | 0.58947 |
| DIO3OS | SIGLEC11 | 1.91E-36 | 0.588734 |
| AP003548.1 | SIGLEC11 | 2.19E-36 | 0.588333 |
| AC104211.2 | SIGLEC11 | 2.37E-36 | 0.58809 |
| ZEB2-AS1 | SIGLEC9 | 2.61E-36 | 0.587813 |
| AC010776.2 | SIGLEC16 | 2.71E-36 | 0.587693 |
| AC138207.5 | CD33 | 3.33E-36 | 0.587084 |
| LINC01150 | SIGLEC7 | 4.09E-36 | 0.586478 |
| AC105094.2 | SIGLEC12 | 4.38E-36 | 0.586271 |
| NAGPA-AS1 | SIGLEC16 | 7.48E-36 | 0.58467 |
| AC136628.1 | SIGLEC11 | 9.11E-36 | 0.584082 |
| MIR223HG | SIGLEC5 | 1.49E-35 | 0.582609 |
| LINC01146 | CD33 | 1.65E-35 | 0.582302 |
| LINC02285 | SIGLEC14 | 1.78E-35 | 0.582063 |
| LINC02391 | SIGLEC9 | 2.47E-35 | 0.581073 |
| AC024337.2 | SIGLEC16 | 3.24E-35 | 0.580247 |
| PIK3CD-AS1 | SIGLEC14 | 3.37E-35 | 0.580127 |
| PCED1B-AS1 | CD33 | 3.82E-35 | 0.57975 |
| LINC02705 | CD33 | 4.61E-35 | 0.579177 |
| AL353611.1 | SIGLEC16 | 8.91E-35 | 0.577155 |
| AC008750.1 | SIGLEC10 | 1.06E-34 | 0.576628 |
| AL355922.1 | SIGLEC12 | 1.26E-34 | 0.576085 |
| LINC01684 | SIGLEC8 | 1.50E-34 | 0.575544 |
| SMIM25 | SIGLEC7 | 1.87E-34 | 0.574868 |
| ZEB2-AS1 | CD33 | 2.05E-34 | 0.574589 |
| AC006033.2 | SIGLEC7 | 2.34E-34 | 0.574171 |
| AC034199.1 | SIGLEC14 | 2.66E-34 | 0.573777 |
| PCED1B-AS1 | SIGLEC14 | 3.38E-34 | 0.573033 |
| AC145098.1 | SIGLEC10 | 4.93E-34 | 0.571851 |
| LINC02705 | SIGLEC9 | 8.89E-34 | 0.569998 |
| AC138207.1 | CD33 | 1.11E-33 | 0.569301 |
| AC245128.3 | SIGLEC7 | 1.21E-33 | 0.569017 |
| LINC01892 | SIGLEC12 | 1.24E-33 | 0.568951 |
| FAM198B-AS1 | SIGLEC11 | 1.26E-33 | 0.568896 |
| AC018755.4 | SIGLEC14 | 1.54E-33 | 0.568272 |
| BX470209.1 | SIGLEC16 | 1.97E-33 | 0.567488 |
| AC008992.1 | SIGLEC11 | 2.21E-33 | 0.567118 |
| PRKAR1B-AS1 | CD33 | 2.47E-33 | 0.566764 |
| AC090559.1 | SIGLEC5 | 2.78E-33 | 0.566381 |
| AC079209.1 | SIGLEC9 | 3.31E-33 | 0.565831 |
| AC027288.3 | SIGLEC11 | 3.44E-33 | 0.565709 |
| LINC00528 | SIGLEC14 | 4.20E-33 | 0.565068 |
| AC034199.1 | SIGLEC5 | 4.21E-33 | 0.565057 |
| AC005692.1 | SIGLEC16 | 5.40E-33 | 0.564259 |
| AC093802.2 | SIGLEC12 | 8.88E-33 | 0.562658 |
| AC243960.1 | SIGLEC8 | 9.44E-33 | 0.562462 |
| AC006033.2 | SIGLEC5 | 1.04E-32 | 0.562158 |
| AC007881.2 | SIGLEC6 | 1.09E-32 | 0.561984 |
| LINC00528 | SIGLEC9 | 1.15E-32 | 0.56182 |
| AC245128.3 | SIGLEC5 | 1.16E-32 | 0.561805 |
| AC007998.3 | SIGLEC11 | 1.22E-32 | 0.561641 |
| LINC01018 | SIGLEC11 | 1.37E-32 | 0.561255 |
| AC011899.3 | SIGLEC9 | 1.77E-32 | 0.56042 |
| AC025279.1 | SIGLEC11 | 1.78E-32 | 0.560412 |
| AC068580.3 | SIGLEC1 | 1.82E-32 | 0.560325 |
| AL133371.2 | CD33 | 2.75E-32 | 0.558989 |
| PRKAR1B-AS1 | SIGLEC14 | 8.27E-32 | 0.555365 |
| CCND2-AS1 | MAG | 9.21E-32 | 0.555008 |
| AC145098.1 | SIGLEC5 | 9.44E-32 | 0.554925 |
| AL133371.2 | SIGLEC1 | 1.72E-31 | 0.552937 |
| AC107958.3 | SIGLEC16 | 1.88E-31 | 0.552629 |
| OBI1-AS1 | SIGLEC12 | 1.91E-31 | 0.552578 |
| AL035670.1 | SIGLEC11 | 2.01E-31 | 0.552416 |
| AC015819.1 | SIGLEC9 | 2.05E-31 | 0.552352 |
| AP001599.1 | SIGLEC11 | 2.21E-31 | 0.552089 |
| PIK3CD-AS1 | CD33 | 2.46E-31 | 0.551738 |
| PDZRN3-AS1 | SIGLEC11 | 2.51E-31 | 0.55167 |
| LINC02642 | SIGLEC14 | 2.83E-31 | 0.551262 |
| LINC01094 | SIGLEC5 | 3.34E-31 | 0.550709 |
| AC004687.1 | CD33 | 4.17E-31 | 0.549963 |
| AC004921.1 | CD33 | 4.85E-31 | 0.54945 |
| AC010776.3 | SIGLEC11 | 5.19E-31 | 0.549224 |
| PRKAR1B-AS1 | SIGLEC7 | 5.85E-31 | 0.548815 |
| AC002128.2 | MAG | 5.88E-31 | 0.5488 |
| HPN-AS1 | MAG | 6.50E-31 | 0.548461 |
| LINC01150 | SIGLEC1 | 7.96E-31 | 0.547771 |
| AC015911.3 | SIGLEC1 | 8.04E-31 | 0.547738 |
| AC055733.2 | SIGLEC11 | 8.86E-31 | 0.54741 |
| AC126773.4 | SIGLEC16 | 1.00E-30 | 0.546992 |
| AC100849.1 | SIGLEC14 | 1.02E-30 | 0.546935 |
| AC004687.1 | SIGLEC1 | 1.11E-30 | 0.546629 |
| LINC00391 | SIGLEC16 | 1.40E-30 | 0.545847 |
| LINC02705 | SIGLEC1 | 1.45E-30 | 0.545738 |
| AC020907.4 | MAG | 1.49E-30 | 0.545627 |
| AC015911.3 | SIGLEC8 | 1.50E-30 | 0.545603 |
| CEP250-AS1 | SIGLEC9 | 2.00E-30 | 0.544629 |
| MMP2-AS1 | SIGLEC14 | 2.10E-30 | 0.544456 |
| AC004381.1 | SIGLEC11 | 2.21E-30 | 0.544291 |
| AC002128.1 | CD22 | 2.31E-30 | 0.544134 |
| AL135960.1 | SIGLEC11 | 2.31E-30 | 0.544132 |
| LINC01094 | SIGLEC8 | 2.41E-30 | 0.543983 |
| AC004921.1 | SIGLEC9 | 3.96E-30 | 0.542272 |
| LINC00924 | SIGLEC16 | 4.05E-30 | 0.542197 |
| GRK5-IT1 | SIGLEC11 | 4.09E-30 | 0.542161 |
| AC079015.1 | SIGLEC8 | 4.63E-30 | 0.541732 |
| AC093583.1 | SIGLEC10 | 5.38E-30 | 0.54121 |
| USP12-AS2 | SIGLEC12 | 5.71E-30 | 0.541005 |
| AL078612.3 | SIGLEC12 | 5.92E-30 | 0.540882 |
| AC068580.3 | SIGLEC9 | 6.37E-30 | 0.540628 |
| AC002101.1 | SIGLEC16 | 6.75E-30 | 0.540423 |
| HAND2-AS1 | SIGLEC11 | 8.23E-30 | 0.539733 |
| AL133371.2 | SIGLEC14 | 9.61E-30 | 0.539193 |
| AC055717.2 | SIGLEC16 | 1.09E-29 | 0.538764 |
| CEP250-AS1 | CD33 | 2.12E-29 | 0.536412 |
| AC010931.1 | SIGLEC16 | 2.26E-29 | 0.536181 |
| AC004687.1 | SIGLEC9 | 2.39E-29 | 0.535993 |
| AC090559.1 | SIGLEC8 | 2.56E-29 | 0.53575 |
| AP001434.1 | SIGLEC10 | 2.63E-29 | 0.535649 |
| AC091806.1 | SIGLEC11 | 3.20E-29 | 0.534953 |
| AL162426.1 | SIGLEC12 | 4.00E-29 | 0.534167 |
| LINC02391 | CD33 | 4.64E-29 | 0.533636 |
| ZEB2-AS1 | SIGLEC1 | 4.99E-29 | 0.533382 |
| LINC01146 | SIGLEC1 | 5.96E-29 | 0.532742 |
| AC138207.4 | SIGLEC1 | 7.76E-29 | 0.5318 |
| AC007728.2 | SIGLEC8 | 7.96E-29 | 0.531708 |
| AC079209.1 | SIGLEC14 | 8.07E-29 | 0.531662 |
| AC007728.2 | SIGLEC1 | 8.15E-29 | 0.531627 |
| AC011467.3 | MAG | 8.19E-29 | 0.531609 |
| LINC02642 | SIGLEC9 | 8.22E-29 | 0.531593 |
| LINC02828 | SIGLEC9 | 1.06E-28 | 0.530676 |
| AC091182.1 | SIGLEC11 | 1.07E-28 | 0.530647 |
| LINC02084 | SIGLEC8 | 1.23E-28 | 0.530138 |
| LINC02147 | SIGLEC11 | 1.69E-28 | 0.528998 |
| AC138207.1 | SIGLEC14 | 1.73E-28 | 0.528906 |
| LINC00528 | CD33 | 2.51E-28 | 0.527563 |
| AC068580.1 | SIGLEC1 | 2.76E-28 | 0.527223 |
| LINC01694 | SIGLEC10 | 2.88E-28 | 0.527071 |
| CEP250-AS1 | SIGLEC14 | 3.13E-28 | 0.526767 |
| AC138207.5 | SIGLEC14 | 4.24E-28 | 0.525652 |
| LINC00498 | SIGLEC12 | 4.27E-28 | 0.52563 |
| AC012409.3 | SIGLEC16 | 4.46E-28 | 0.525467 |
| LINC02269 | SIGLEC11 | 5.77E-28 | 0.524527 |
| AC245128.3 | SIGLEC14 | 6.06E-28 | 0.524346 |
| MEF2C-AS1 | SIGLEC16 | 7.24E-28 | 0.523692 |
| AC027013.1 | SIGLEC11 | 7.49E-28 | 0.523571 |
| AC105219.3 | SIGLEC12 | 8.03E-28 | 0.523311 |
| AC093583.1 | SIGLEC9 | 9.17E-28 | 0.522823 |
| AC034199.1 | SIGLEC9 | 1.00E-27 | 0.5225 |
| AC007923.1 | MAG | 1.04E-27 | 0.522343 |
| AC015819.1 | CD33 | 1.44E-27 | 0.521144 |
| LINC01934 | SIGLEC8 | 1.69E-27 | 0.52055 |
| AC068580.1 | SIGLEC9 | 1.76E-27 | 0.520401 |
| AC138207.1 | SIGLEC7 | 1.85E-27 | 0.520227 |
| AL445490.1 | SIGLEC1 | 2.05E-27 | 0.519838 |
| LINC02528 | SIGLEC14 | 2.28E-27 | 0.519441 |
| AC079209.1 | CD33 | 2.56E-27 | 0.519011 |
| LINC02622 | SIGLEC6 | 2.69E-27 | 0.518829 |
| SMIM25 | CD33 | 2.71E-27 | 0.518795 |
| FAM41C | SIGLEC6 | 2.85E-27 | 0.518612 |
| AC099328.2 | MAG | 4.20E-27 | 0.517153 |
| AC011899.3 | SIGLEC14 | 4.40E-27 | 0.516978 |
| AL590764.1 | SIGLEC8 | 4.71E-27 | 0.516726 |
| LINC00528 | SIGLEC1 | 6.47E-27 | 0.515523 |
| ADPGK-AS1 | SIGLEC1 | 6.53E-27 | 0.515491 |
| AC004687.1 | SIGLEC14 | 7.60E-27 | 0.514916 |
| AC012181.1 | SIGLEC1 | 9.36E-27 | 0.514126 |
| AL158042.1 | SIGLEC16 | 9.37E-27 | 0.514122 |
| MIR503HG | SIGLEC11 | 1.10E-26 | 0.513525 |
| LINC00426 | SIGLEC10 | 1.15E-26 | 0.513327 |
| LINC02828 | CD33 | 1.21E-26 | 0.513156 |
| AC063919.1 | MAG | 1.58E-26 | 0.512133 |
| MMP2-AS1 | SIGLEC9 | 2.17E-26 | 0.51091 |
| CCR5AS | SIGLEC5 | 2.26E-26 | 0.51076 |
| AC023154.1 | SIGLEC16 | 2.47E-26 | 0.51042 |
| AP002954.1 | SIGLEC9 | 3.54E-26 | 0.509028 |
| PCED1B-AS1 | SIGLEC7 | 3.67E-26 | 0.508889 |
| AC004946.2 | SIGLEC12 | 3.81E-26 | 0.508744 |
| LINC01150 | SIGLEC8 | 4.58E-26 | 0.508033 |
| AC110995.1 | SIGLEC8 | 4.60E-26 | 0.508013 |
| AC020907.2 | MAG | 5.67E-26 | 0.507208 |
| AP001610.2 | SIGLEC1 | 5.99E-26 | 0.506991 |
| LINC02642 | SIGLEC1 | 7.81E-26 | 0.505956 |
| LINC01391 | SIGLEC16 | 8.03E-26 | 0.505848 |
| AC011899.2 | SIGLEC8 | 8.43E-26 | 0.505661 |
| AL133371.2 | SIGLEC7 | 1.02E-25 | 0.504912 |
| LINC01197 | SIGLEC16 | 1.02E-25 | 0.504908 |
| AC079209.1 | SIGLEC7 | 1.24E-25 | 0.50414 |
| AC245128.3 | CD33 | 1.26E-25 | 0.504082 |
| AC011899.3 | CD33 | 1.35E-25 | 0.503827 |
| C9orf139 | SIGLEC9 | 1.57E-25 | 0.503213 |
| LINC01798 | SIGLEC15 | 1.64E-25 | 0.503047 |
| AC092155.1 | SIGLEC16 | 1.86E-25 | 0.502552 |
| LINC01994 | SIGLEC16 | 2.15E-25 | 0.501977 |
| AC012181.2 | SIGLEC1 | 2.17E-25 | 0.501944 |
| SMIM25 | SIGLEC14 | 2.33E-25 | 0.501671 |
| NR2F2-AS1 | SIGLEC16 | 2.49E-25 | 0.501401 |
| AC011899.2 | SIGLEC10 | 2.93E-25 | 0.500756 |
| AC068580.3 | SIGLEC7 | 3.47E-25 | 0.500087 |
| AL021707.3 | SIGLEC1 | 3.56E-25 | 0.499976 |
| AC138207.5 | SIGLEC1 | 3.99E-25 | 0.499526 |
| LINC01684 | SIGLEC14 | 4.99E-25 | 0.49863 |
| PSMB8-AS1 | SIGLEC14 | 5.87E-25 | 0.497987 |
| AC244453.1 | SIGLEC11 | 6.53E-25 | 0.497554 |
| AL360267.1 | SIGLEC12 | 7.42E-25 | 0.497041 |
| AC011899.3 | SIGLEC12 | 7.65E-25 | 0.496919 |
| LINC00426 | SIGLEC8 | 8.37E-25 | 0.496558 |
| AL731557.1 | SIGLEC11 | 8.47E-25 | 0.496512 |
| MMP2-AS1 | SIGLEC1 | 9.38E-25 | 0.496102 |
| MIR202HG | SIGLEC16 | 1.02E-24 | 0.495772 |
| AC108010.1 | SIGLEC1 | 1.37E-24 | 0.494579 |
| AC090559.1 | SIGLEC10 | 1.37E-24 | 0.494566 |
| AL133371.2 | SIGLEC8 | 1.53E-24 | 0.49412 |
| GRTP1-AS1 | SIGLEC16 | 1.56E-24 | 0.494039 |
| AC021752.1 | SIGLEC12 | 1.58E-24 | 0.493999 |
| MIR223HG | SIGLEC9 | 1.79E-24 | 0.493481 |
| AC090213.1 | SIGLEC16 | 1.99E-24 | 0.493061 |
| AC012409.2 | SIGLEC11 | 1.99E-24 | 0.493044 |
| AC008992.1 | SIGLEC16 | 2.02E-24 | 0.492991 |
| Z99756.1 | SIGLEC16 | 2.07E-24 | 0.492894 |
| TRG-AS1 | SIGLEC9 | 2.09E-24 | 0.492861 |
| AC105254.1 | SIGLEC11 | 2.12E-24 | 0.492795 |
| AC007728.2 | SIGLEC9 | 2.13E-24 | 0.492782 |
| AC034199.1 | SIGLEC7 | 2.46E-24 | 0.492181 |
| AC138207.5 | SIGLEC8 | 2.59E-24 | 0.491977 |
| PIK3CD-AS1 | SIGLEC8 | 2.67E-24 | 0.491851 |
| AC079015.1 | SIGLEC14 | 2.68E-24 | 0.491837 |
| PIK3CD-AS1 | SIGLEC7 | 2.78E-24 | 0.491688 |
| AC092484.1 | SIGLEC10 | 3.07E-24 | 0.491278 |
| ZNF30-AS1 | CD22 | 3.28E-24 | 0.491013 |
| AC002128.2 | CD22 | 3.83E-24 | 0.490373 |
| AC100849.1 | SIGLEC7 | 4.02E-24 | 0.49018 |
| AC022166.1 | SIGLEC16 | 4.62E-24 | 0.489604 |
| AC004585.1 | SIGLEC7 | 5.19E-24 | 0.489125 |
| AL021153.1 | SIGLEC11 | 5.25E-24 | 0.489077 |
| C8orf49 | SIGLEC16 | 5.33E-24 | 0.489015 |
| TRG-AS1 | CD33 | 5.58E-24 | 0.488827 |
| LINC02528 | SIGLEC7 | 6.01E-24 | 0.488518 |
| AC083862.1 | SIGLEC1 | 6.06E-24 | 0.488483 |
| AC034238.2 | SIGLEC8 | 6.14E-24 | 0.488434 |
| AC010636.1 | SIGLEC16 | 6.20E-24 | 0.488389 |
| MACORIS | CD33 | 6.89E-24 | 0.487955 |
| PTPRD-AS1 | SIGLEC11 | 7.88E-24 | 0.487397 |
| C4A-AS1 | SIGLEC12 | 8.83E-24 | 0.486925 |
| AC099524.1 | SIGLEC14 | 9.67E-24 | 0.486545 |
| AC006272.1 | SIGLEC9 | 1.05E-23 | 0.486214 |
| AC078923.1 | SIGLEC16 | 1.05E-23 | 0.486207 |
| AL590385.2 | SIGLEC5 | 1.15E-23 | 0.485822 |
| AC012181.2 | SIGLEC9 | 1.32E-23 | 0.485251 |
| LINC01606 | SIGLEC6 | 1.64E-23 | 0.484344 |
| AL162414.1 | SIGLEC7 | 1.75E-23 | 0.484066 |
| AC012085.2 | SIGLEC11 | 1.85E-23 | 0.483847 |
| CARD8-AS1 | SIGLEC7 | 1.98E-23 | 0.48356 |
| AC120498.8 | SIGLEC11 | 1.99E-23 | 0.483536 |
| MIR223HG | CD33 | 2.09E-23 | 0.483329 |
| AC008957.1 | CD33 | 2.42E-23 | 0.482705 |
| AC006064.1 | SIGLEC1 | 2.64E-23 | 0.482336 |
| LINC01146 | SIGLEC7 | 2.82E-23 | 0.482057 |
| PIK3CD-AS1 | SIGLEC5 | 3.12E-23 | 0.481639 |
| AC015819.1 | SIGLEC14 | 3.12E-23 | 0.481633 |
| C9orf147 | SIGLEC11 | 3.21E-23 | 0.481514 |
| AC091820.2 | SIGLEC16 | 3.35E-23 | 0.481341 |
| AC015819.1 | SIGLEC7 | 3.85E-23 | 0.480743 |
| PABPC5-AS1 | SIGLEC11 | 3.95E-23 | 0.480638 |
| LINC00861 | SIGLEC8 | 4.15E-23 | 0.480427 |
| LINC00528 | SIGLEC7 | 4.53E-23 | 0.480056 |
| AC100849.1 | CD33 | 4.81E-23 | 0.479804 |
| AC090796.1 | SIGLEC12 | 5.00E-23 | 0.479639 |
| LINC01262 | SIGLEC16 | 5.45E-23 | 0.479269 |
| BX255923.1 | SIGLEC11 | 5.73E-23 | 0.479059 |
| AC073311.1 | SIGLEC16 | 6.40E-23 | 0.478582 |
| TRG-AS1 | SIGLEC14 | 7.36E-23 | 0.477988 |
| AP002954.1 | SIGLEC14 | 9.13E-23 | 0.477064 |
| AC093583.1 | CD33 | 9.73E-23 | 0.476791 |
| AC006305.4 | SIGLEC16 | 9.96E-23 | 0.476688 |
| AL049775.1 | SIGLEC11 | 9.97E-23 | 0.476683 |
| AC004551.1 | SIGLEC1 | 1.06E-22 | 0.47641 |
| AC020907.4 | CD22 | 1.27E-22 | 0.475632 |
| CCR5AS | SIGLEC8 | 1.39E-22 | 0.475239 |
| CCR5AS | SIGLEC1 | 1.45E-22 | 0.475076 |
| MMP2-AS1 | SIGLEC8 | 1.46E-22 | 0.475042 |
| ZEB2-AS1 | SIGLEC14 | 1.72E-22 | 0.474322 |
| AL035530.1 | SIGLEC9 | 1.85E-22 | 0.474011 |
| AL139351.1 | SIGLEC16 | 1.88E-22 | 0.473952 |
| TARID | SIGLEC11 | 2.33E-22 | 0.473019 |
| LINC01684 | SIGLEC9 | 2.39E-22 | 0.472901 |
| AC004921.1 | SIGLEC14 | 2.64E-22 | 0.472475 |
| AC068944.1 | SIGLEC16 | 3.32E-22 | 0.471475 |
| CCR5AS | SIGLEC10 | 3.35E-22 | 0.471438 |
| AL450998.3 | SIGLEC1 | 4.12E-22 | 0.470523 |
| LINC02528 | SIGLEC9 | 4.55E-22 | 0.470091 |
| MIR223HG | SIGLEC14 | 5.16E-22 | 0.469536 |
| AL121827.2 | SIGLEC6 | 5.27E-22 | 0.469446 |
| ERC2-IT1 | SIGLEC16 | 5.48E-22 | 0.469274 |
| AC093583.1 | SIGLEC7 | 5.78E-22 | 0.469037 |
| AP002954.1 | CD33 | 6.37E-22 | 0.46861 |
| AC002398.2 | SIGLEC6 | 6.38E-22 | 0.468606 |
| LINC02391 | SIGLEC14 | 6.70E-22 | 0.468391 |
| AC079921.1 | SIGLEC1 | 7.67E-22 | 0.467791 |
| AC007040.1 | SIGLEC6 | 8.02E-22 | 0.467595 |
| LINC02391 | SIGLEC7 | 8.26E-22 | 0.467462 |
| AC004522.4 | MAG | 8.79E-22 | 0.467185 |
| AC068580.1 | SIGLEC7 | 9.50E-22 | 0.46684 |
| AC009292.1 | MAG | 9.63E-22 | 0.466782 |
| AL731567.1 | SIGLEC9 | 1.01E-21 | 0.46658 |
| AL590867.1 | MAG | 1.04E-21 | 0.466451 |
| AC145098.1 | SIGLEC8 | 1.09E-21 | 0.46623 |
| LINC01679 | SIGLEC1 | 1.12E-21 | 0.466125 |
| ADPGK-AS1 | SIGLEC9 | 1.14E-21 | 0.466046 |
| AC073346.1 | SIGLEC12 | 1.16E-21 | 0.465939 |
| AC083862.1 | SIGLEC14 | 1.17E-21 | 0.465915 |
| AC006947.1 | SIGLEC12 | 1.25E-21 | 0.465616 |
| C9orf139 | CD33 | 1.29E-21 | 0.465477 |
| LINC01797 | SIGLEC15 | 1.30E-21 | 0.465462 |
| AL353614.1 | SIGLEC16 | 1.41E-21 | 0.465078 |
| AC093801.1 | SIGLEC10 | 1.45E-21 | 0.464953 |
| LINC01094 | SIGLEC10 | 1.65E-21 | 0.464395 |
| AC090771.2 | SIGLEC12 | 1.84E-21 | 0.463904 |
| LINC01679 | SIGLEC7 | 1.88E-21 | 0.463798 |
| DIO3OS | SIGLEC16 | 1.88E-21 | 0.463792 |
| LINC02446 | SIGLEC8 | 1.91E-21 | 0.463724 |
| LINC01727 | MAG | 1.93E-21 | 0.463685 |
| AC008840.1 | SIGLEC9 | 1.95E-21 | 0.463643 |
| LINC01679 | SIGLEC9 | 2.04E-21 | 0.463421 |
| LINC02553 | MAG | 2.09E-21 | 0.463314 |
| CASC18 | MAG | 2.75E-21 | 0.462093 |
| AP001172.1 | SIGLEC11 | 2.75E-21 | 0.462088 |
| AC006272.1 | SIGLEC1 | 2.83E-21 | 0.461961 |
| AC015819.1 | SIGLEC1 | 3.03E-21 | 0.461656 |
| CARD8-AS1 | SIGLEC9 | 3.06E-21 | 0.461608 |
| AC006369.1 | SIGLEC8 | 3.07E-21 | 0.461586 |
| LINC01947 | SIGLEC16 | 3.15E-21 | 0.461472 |
| TRG-AS1 | SIGLEC7 | 3.74E-21 | 0.460701 |
| AC015911.3 | SIGLEC14 | 3.94E-21 | 0.460458 |
| AC245884.11 | SIGLEC5 | 3.99E-21 | 0.460401 |
| C9orf139 | SIGLEC1 | 4.53E-21 | 0.459829 |
| AC138207.4 | SIGLEC14 | 4.70E-21 | 0.459664 |
| MROCKI | SIGLEC1 | 4.93E-21 | 0.459443 |
| AC078820.2 | SIGLEC16 | 5.15E-21 | 0.459247 |
| AC008957.1 | SIGLEC9 | 5.23E-21 | 0.45918 |
| AL590385.2 | SIGLEC1 | 5.56E-21 | 0.458899 |
| AL022318.1 | SIGLEC6 | 6.14E-21 | 0.458448 |
| LINC01909 | SIGLEC16 | 6.25E-21 | 0.458365 |
| AC011899.3 | SIGLEC7 | 6.27E-21 | 0.458354 |
| AL596451.1 | SIGLEC12 | 6.97E-21 | 0.457868 |
| LINC01684 | SIGLEC1 | 7.20E-21 | 0.457718 |
| AL591222.1 | SIGLEC6 | 7.35E-21 | 0.457625 |
| LINC01150 | SIGLEC5 | 7.46E-21 | 0.457559 |
| CARD8-AS1 | CD33 | 7.60E-21 | 0.457469 |
| AC010731.3 | MAG | 7.84E-21 | 0.457328 |
| AL390783.1 | SIGLEC11 | 7.85E-21 | 0.457324 |
| AC004687.1 | SIGLEC8 | 7.96E-21 | 0.457258 |
| AC243960.1 | SIGLEC14 | 8.15E-21 | 0.457152 |
| AC003070.1 | SIGLEC1 | 8.63E-21 | 0.456887 |
| LINC02360 | SIGLEC16 | 9.23E-21 | 0.45658 |
| HCP5 | SIGLEC14 | 9.39E-21 | 0.456504 |
| AC092484.1 | SIGLEC12 | 9.66E-21 | 0.456371 |
| ZEB2-AS1 | SIGLEC7 | 9.76E-21 | 0.456324 |
| PCED1B-AS1 | SIGLEC8 | 1.00E-20 | 0.45621 |
| AC107204.1 | MAG | 1.05E-20 | 0.455984 |
| AC006272.1 | SIGLEC14 | 1.07E-20 | 0.455901 |
| AL121768.1 | SIGLEC11 | 1.14E-20 | 0.455618 |
| AC006450.2 | SIGLEC16 | 1.32E-20 | 0.454945 |
| PSMB8-AS1 | SIGLEC8 | 1.33E-20 | 0.454914 |
| LINC02099 | SIGLEC8 | 1.34E-20 | 0.454851 |
| AC110995.1 | SIGLEC5 | 1.37E-20 | 0.454756 |
| AC016910.2 | SIGLEC11 | 1.38E-20 | 0.454716 |
| AL355922.1 | SIGLEC9 | 1.42E-20 | 0.454586 |
| AC064807.2 | SIGLEC6 | 1.44E-20 | 0.454548 |
| LINC01679 | SIGLEC14 | 1.49E-20 | 0.454379 |
| AL683807.1 | SIGLEC8 | 1.53E-20 | 0.454243 |
| AC015540.1 | SIGLEC11 | 1.69E-20 | 0.45379 |
| LINC02418 | SIGLEC16 | 1.70E-20 | 0.453777 |
| LINC01907 | SIGLEC1 | 1.90E-20 | 0.453257 |
| LINC01907 | SIGLEC9 | 1.98E-20 | 0.453064 |
| AC090796.1 | SIGLEC9 | 2.16E-20 | 0.452652 |
| HLA-DQB1-AS1 | SIGLEC8 | 2.21E-20 | 0.452545 |
| AC063944.3 | SIGLEC11 | 2.22E-20 | 0.452533 |
| MMP2-AS1 | SIGLEC10 | 2.41E-20 | 0.45214 |
| AC138207.5 | SIGLEC7 | 2.45E-20 | 0.452073 |
| AC012181.2 | CD33 | 2.63E-20 | 0.451731 |
| MMP2-AS1 | SIGLEC5 | 2.70E-20 | 0.451607 |
| AL391832.3 | SIGLEC9 | 2.76E-20 | 0.451513 |
| AL391832.3 | CD33 | 2.90E-20 | 0.451286 |
| AC025175.1 | SIGLEC11 | 2.99E-20 | 0.451131 |
| AC120498.10 | SIGLEC16 | 3.03E-20 | 0.451073 |
| LINC02176 | SIGLEC11 | 3.04E-20 | 0.451062 |
| AC138207.1 | SIGLEC5 | 3.07E-20 | 0.45102 |
| AL035530.1 | SIGLEC14 | 3.15E-20 | 0.450898 |
| AC243829.4 | SIGLEC8 | 3.15E-20 | 0.450892 |
| AL445493.3 | SIGLEC11 | 3.43E-20 | 0.450499 |
| AC011377.1 | SIGLEC1 | 3.45E-20 | 0.450472 |
| LINC02705 | SIGLEC7 | 3.68E-20 | 0.45017 |
| AL049840.5 | SIGLEC1 | 3.73E-20 | 0.450103 |
| AC092145.1 | SIGLEC1 | 3.79E-20 | 0.450024 |
| AC245884.11 | SIGLEC1 | 5.71E-20 | 0.448097 |
| LINC02528 | SIGLEC8 | 6.03E-20 | 0.447843 |
| AC243960.1 | SIGLEC9 | 6.16E-20 | 0.447741 |
| SMIM25 | SIGLEC5 | 6.62E-20 | 0.447401 |
| LINC00996 | SIGLEC14 | 7.16E-20 | 0.447028 |
| ZEB2-AS1 | SIGLEC8 | 7.20E-20 | 0.447005 |
| AL590385.2 | SIGLEC9 | 8.02E-20 | 0.44649 |
| PCED1B-AS1 | SIGLEC12 | 8.45E-20 | 0.446242 |
| LINC00528 | SIGLEC5 | 8.98E-20 | 0.445955 |
| AC022126.1 | SIGLEC8 | 9.08E-20 | 0.445905 |
| AC020892.2 | SIGLEC16 | 9.17E-20 | 0.445853 |
| CARD8-AS1 | SIGLEC14 | 9.60E-20 | 0.445636 |
| AC093895.2 | SIGLEC9 | 1.02E-19 | 0.445353 |
| TMSB15B-AS1 | SIGLEC11 | 1.06E-19 | 0.445153 |
| AC009950.1 | SIGLEC1 | 1.07E-19 | 0.445103 |
| LINC01799 | SIGLEC15 | 1.10E-19 | 0.445003 |
| LINC01871 | SIGLEC8 | 1.10E-19 | 0.444995 |
| AC243960.1 | CD33 | 1.24E-19 | 0.444421 |
| AC092484.1 | CD33 | 1.27E-19 | 0.444305 |
| AC008601.1 | SIGLEC16 | 1.27E-19 | 0.444302 |
| LINC02040 | SIGLEC11 | 1.28E-19 | 0.444266 |
| AL590764.1 | SIGLEC1 | 1.32E-19 | 0.444122 |
| PRKAR1B-AS1 | SIGLEC1 | 1.41E-19 | 0.443819 |
| TRG-AS1 | SIGLEC1 | 1.41E-19 | 0.443801 |
| LINC02642 | SIGLEC8 | 1.53E-19 | 0.443421 |
| LINC00670 | SIGLEC11 | 1.55E-19 | 0.443338 |
| CEP250-AS1 | SIGLEC7 | 1.60E-19 | 0.443189 |
| AC034238.2 | SIGLEC14 | 1.69E-19 | 0.442948 |
| AC068580.3 | SIGLEC14 | 1.70E-19 | 0.442912 |
| LINC02285 | SIGLEC1 | 1.81E-19 | 0.442617 |
| AC138028.4 | SIGLEC1 | 1.83E-19 | 0.442543 |
| CARMN | SIGLEC16 | 1.86E-19 | 0.442485 |
| AC121247.2 | SIGLEC11 | 1.86E-19 | 0.442474 |
| AC114811.2 | SIGLEC11 | 1.97E-19 | 0.442201 |
| AL162414.1 | SIGLEC14 | 1.97E-19 | 0.442192 |
| MRPL23-AS1 | SIGLEC6 | 2.01E-19 | 0.442108 |
| AC093278.2 | SIGLEC1 | 2.01E-19 | 0.4421 |
| LINC02121 | SIGLEC11 | 2.07E-19 | 0.44195 |
| AC004921.1 | SIGLEC7 | 2.10E-19 | 0.441888 |
| MMP2-AS1 | CD33 | 2.11E-19 | 0.44186 |
| ADPGK-AS1 | SIGLEC7 | 2.36E-19 | 0.441334 |
| AC087477.5 | SIGLEC16 | 2.42E-19 | 0.44121 |
| LINC00708 | SIGLEC16 | 2.52E-19 | 0.441017 |
| LINC02705 | SIGLEC14 | 2.67E-19 | 0.440738 |
| CLYBL-AS1 | MAG | 2.73E-19 | 0.440623 |
| AL133371.2 | SIGLEC5 | 2.78E-19 | 0.44054 |
| AL731567.1 | CD33 | 2.82E-19 | 0.440462 |
| AC012181.1 | SIGLEC5 | 3.17E-19 | 0.439893 |
| MROCKI | CD33 | 3.22E-19 | 0.439823 |
| NFE4 | SIGLEC16 | 3.24E-19 | 0.439789 |
| AC097532.1 | SIGLEC7 | 3.25E-19 | 0.439782 |
| AP000695.1 | SIGLEC5 | 3.58E-19 | 0.439309 |
| AC006064.1 | SIGLEC8 | 3.91E-19 | 0.438875 |
| AL031846.2 | SIGLEC1 | 4.02E-19 | 0.438741 |
| AC079209.1 | SIGLEC5 | 4.08E-19 | 0.438671 |
| SENCR | SIGLEC10 | 4.35E-19 | 0.438357 |
| AL731567.1 | SIGLEC14 | 4.50E-19 | 0.438195 |
| AC015819.1 | SIGLEC8 | 4.82E-19 | 0.437864 |
| AL162414.1 | SIGLEC9 | 4.86E-19 | 0.437823 |
| AP005242.1 | SIGLEC11 | 5.09E-19 | 0.43759 |
| AC025809.1 | SIGLEC11 | 5.18E-19 | 0.437513 |
| AC245884.11 | SIGLEC14 | 5.46E-19 | 0.43725 |
| AP002954.1 | SIGLEC7 | 5.74E-19 | 0.43701 |
| LINC00528 | SIGLEC8 | 5.99E-19 | 0.436799 |
| AC093334.1 | SIGLEC11 | 6.01E-19 | 0.436781 |
| AC099524.1 | SIGLEC9 | 6.21E-19 | 0.436623 |
| AP000695.1 | SIGLEC9 | 6.30E-19 | 0.436553 |
| LINC01898 | SIGLEC15 | 6.33E-19 | 0.436525 |
| MROCKI | SIGLEC14 | 6.39E-19 | 0.436483 |
| AC007728.2 | SIGLEC7 | 6.92E-19 | 0.436092 |
| AC092484.1 | SIGLEC9 | 7.03E-19 | 0.436011 |
| AC079015.1 | SIGLEC1 | 7.37E-19 | 0.43578 |
| AC008750.1 | SIGLEC14 | 7.47E-19 | 0.435713 |
| AC022034.4 | SIGLEC11 | 8.33E-19 | 0.435179 |
| Z82214.1 | SIGLEC11 | 8.40E-19 | 0.435136 |
| GSN-AS1 | SIGLEC1 | 8.63E-19 | 0.435 |
| LINC00955 | SIGLEC7 | 9.74E-19 | 0.434404 |
| AC092667.1 | SIGLEC11 | 1.06E-18 | 0.433978 |
| AC006272.1 | SIGLEC7 | 1.10E-18 | 0.433812 |
| C1orf147 | SIGLEC1 | 1.13E-18 | 0.433693 |
| AC018755.4 | SIGLEC9 | 1.15E-18 | 0.433594 |
| AC018755.4 | CD33 | 1.21E-18 | 0.433325 |
| AC079015.1 | SIGLEC9 | 1.32E-18 | 0.432912 |
| AC060234.2 | SIGLEC11 | 1.34E-18 | 0.43281 |
| AC079414.1 | SIGLEC7 | 1.40E-18 | 0.432625 |
| AC008759.3 | SIGLEC8 | 1.49E-18 | 0.432315 |
| TNK2-AS1 | SIGLEC1 | 1.57E-18 | 0.432052 |
| AC027804.1 | SIGLEC11 | 1.61E-18 | 0.431912 |
| AC008750.1 | SIGLEC9 | 1.65E-18 | 0.431788 |
| AC010519.1 | SIGLEC11 | 1.66E-18 | 0.431771 |
| LINC00996 | CD33 | 1.76E-18 | 0.431471 |
| AC004817.3 | SIGLEC1 | 1.78E-18 | 0.431405 |
| AC099524.1 | SIGLEC8 | 1.80E-18 | 0.431363 |
| AC034199.1 | SIGLEC10 | 1.81E-18 | 0.43134 |
| AL683807.1 | SIGLEC1 | 1.83E-18 | 0.431278 |
| HPN-AS1 | CD22 | 1.87E-18 | 0.431178 |
| LEMD1-AS1 | SIGLEC11 | 2.04E-18 | 0.430743 |
| AC012181.2 | SIGLEC7 | 2.05E-18 | 0.430701 |
| LINC02773 | SIGLEC14 | 2.09E-18 | 0.430605 |
| AC138207.1 | SIGLEC8 | 2.18E-18 | 0.430395 |
| AC068580.1 | SIGLEC14 | 2.19E-18 | 0.430389 |
| LINC02101 | SIGLEC11 | 2.22E-18 | 0.430312 |
| CCND2-AS1 | CD22 | 2.27E-18 | 0.430196 |
| HCG22 | MAG | 2.31E-18 | 0.43011 |
| AC079209.1 | SIGLEC1 | 2.35E-18 | 0.430034 |
| AC034199.1 | CD33 | 2.36E-18 | 0.430012 |
| FP325335.1 | SIGLEC6 | 2.41E-18 | 0.429906 |
| AC078788.1 | SIGLEC8 | 2.48E-18 | 0.429759 |
| AC079015.1 | CD33 | 2.90E-18 | 0.428974 |
| AC012181.1 | SIGLEC9 | 2.96E-18 | 0.42886 |
| AC026124.1 | SIGLEC16 | 3.11E-18 | 0.428619 |
| AC022166.2 | SIGLEC11 | 3.23E-18 | 0.428425 |
| MMP2-AS1 | SIGLEC7 | 3.32E-18 | 0.428281 |
| AC099524.1 | CD33 | 3.50E-18 | 0.428023 |
| AL392183.1 | SIGLEC12 | 3.59E-18 | 0.427892 |
| AC093583.1 | SIGLEC14 | 3.79E-18 | 0.427626 |
| AC125613.1 | SIGLEC16 | 3.81E-18 | 0.427595 |
| AC006064.1 | SIGLEC9 | 3.83E-18 | 0.427571 |
| LINC02642 | CD33 | 3.93E-18 | 0.427433 |
| AC093278.2 | SIGLEC9 | 4.00E-18 | 0.427345 |
| LINC01146 | SIGLEC5 | 4.02E-18 | 0.427319 |
| ADAMTS9-AS2 | SIGLEC16 | 4.07E-18 | 0.42726 |
| AC084064.1 | SIGLEC16 | 4.21E-18 | 0.427086 |
| GNA14-AS1 | SIGLEC11 | 4.46E-18 | 0.426799 |
| AC005330.1 | SIGLEC6 | 4.73E-18 | 0.426502 |
| AL731567.1 | SIGLEC1 | 4.82E-18 | 0.426403 |
| AC007728.2 | CD33 | 4.95E-18 | 0.426265 |
| LINC02828 | SIGLEC14 | 5.07E-18 | 0.426151 |
| SALRNA1 | SIGLEC15 | 5.21E-18 | 0.426007 |
| AC084048.1 | SIGLEC8 | 5.22E-18 | 0.425996 |
| AC005180.1 | SIGLEC11 | 5.35E-18 | 0.425878 |
| AC121154.1 | SIGLEC16 | 5.45E-18 | 0.425784 |
| AC078850.1 | SIGLEC9 | 5.46E-18 | 0.425773 |
| AC018529.2 | SIGLEC1 | 5.92E-18 | 0.425357 |
| AL035530.1 | SIGLEC7 | 5.94E-18 | 0.425346 |
| AC093278.2 | SIGLEC11 | 6.07E-18 | 0.425228 |
| AC048382.4 | SIGLEC11 | 6.28E-18 | 0.425059 |
| LINC02207 | SIGLEC5 | 6.52E-18 | 0.424869 |
| LINC02773 | SIGLEC9 | 7.02E-18 | 0.424493 |
| LINC00426 | SIGLEC14 | 7.22E-18 | 0.42435 |
| MROCKI | SIGLEC9 | 7.27E-18 | 0.42431 |
| LINC02694 | SIGLEC9 | 7.71E-18 | 0.424012 |
| AC006305.4 | SIGLEC11 | 8.38E-18 | 0.423584 |
| LINC00861 | SIGLEC1 | 8.92E-18 | 0.423267 |
| LINC00996 | SIGLEC8 | 9.12E-18 | 0.423151 |
| AC011726.3 | SIGLEC16 | 9.62E-18 | 0.422878 |
| AC003986.1 | MAG | 9.65E-18 | 0.422864 |
| AC120498.8 | SIGLEC16 | 9.71E-18 | 0.422832 |
| AC068580.3 | CD33 | 9.82E-18 | 0.422772 |
| AC022915.2 | SIGLEC11 | 1.09E-17 | 0.422255 |
| AC007278.1 | SIGLEC1 | 1.15E-17 | 0.421941 |
| LINC02391 | SIGLEC1 | 1.16E-17 | 0.421907 |
| AC007336.2 | CD33 | 1.21E-17 | 0.421704 |
| AL008726.1 | SIGLEC1 | 1.23E-17 | 0.421609 |
| AC010247.1 | SIGLEC9 | 1.24E-17 | 0.42156 |
| LINC01943 | SIGLEC7 | 1.36E-17 | 0.421105 |
| PRR34 | SIGLEC9 | 1.59E-17 | 0.420301 |
| AC007344.1 | MAG | 1.62E-17 | 0.4202 |
| GK-AS1 | SIGLEC1 | 1.62E-17 | 0.420192 |
| AC004585.1 | SIGLEC9 | 1.65E-17 | 0.420093 |
| AC006033.2 | SIGLEC10 | 1.67E-17 | 0.42002 |
| AP002954.1 | SIGLEC5 | 1.76E-17 | 0.419764 |
| IL10RB-DT | SIGLEC9 | 1.77E-17 | 0.41972 |
| CLEC12A-AS1 | SIGLEC9 | 1.79E-17 | 0.419688 |
| AC243960.1 | SIGLEC7 | 1.87E-17 | 0.419462 |
| AC012103.1 | SIGLEC10 | 1.93E-17 | 0.419291 |
| AC008147.2 | SIGLEC1 | 1.94E-17 | 0.419254 |
| AL136320.1 | SIGLEC1 | 1.98E-17 | 0.419163 |
| FAM198B-AS1 | SIGLEC9 | 2.05E-17 | 0.418976 |
| AC025279.1 | SIGLEC16 | 2.12E-17 | 0.418788 |
| LINC02828 | SIGLEC7 | 2.17E-17 | 0.418668 |
| AL139008.2 | SIGLEC12 | 2.30E-17 | 0.418383 |
| LINC01684 | CD33 | 2.33E-17 | 0.4183 |
| AL031600.1 | SIGLEC1 | 2.63E-17 | 0.417671 |
| LINC02785 | SIGLEC9 | 2.66E-17 | 0.417611 |
| AC092376.2 | SIGLEC11 | 2.73E-17 | 0.417486 |
| LINC02157 | SIGLEC11 | 2.79E-17 | 0.417356 |
| AC008840.1 | SIGLEC1 | 3.00E-17 | 0.416978 |
| AC137932.3 | SIGLEC1 | 3.14E-17 | 0.416746 |
| EGFLAM-AS4 | SIGLEC6 | 3.19E-17 | 0.416669 |
| AC015911.3 | SIGLEC9 | 3.28E-17 | 0.416523 |
| AC004381.1 | SIGLEC16 | 3.40E-17 | 0.416327 |
| AC004585.1 | SIGLEC14 | 3.58E-17 | 0.416058 |
| AL022328.3 | SIGLEC1 | 4.01E-17 | 0.415464 |
| AC008105.3 | SIGLEC1 | 4.62E-17 | 0.414715 |
| LINC00892 | SIGLEC14 | 4.63E-17 | 0.414704 |
| AC063919.1 | CD22 | 4.98E-17 | 0.41432 |
| AC104260.1 | SIGLEC16 | 5.01E-17 | 0.414287 |
| AC109460.3 | SIGLEC1 | 5.35E-17 | 0.413944 |
| AC008083.4 | SIGLEC11 | 5.49E-17 | 0.413811 |
| CELF2-AS1 | SIGLEC10 | 5.54E-17 | 0.413755 |
| MRGPRF-AS1 | SIGLEC16 | 5.74E-17 | 0.413575 |
| AC009686.2 | SIGLEC12 | 6.10E-17 | 0.413249 |
| LINC00996 | SIGLEC7 | 6.27E-17 | 0.413106 |
| AC093110.1 | SIGLEC11 | 6.28E-17 | 0.413091 |
| C9orf139 | SIGLEC14 | 6.42E-17 | 0.412975 |
| LINC00996 | SIGLEC9 | 6.46E-17 | 0.412945 |
| AL049840.4 | SIGLEC1 | 6.69E-17 | 0.412755 |
| AC068580.3 | SIGLEC5 | 7.03E-17 | 0.412495 |
| AC079209.1 | SIGLEC8 | 7.05E-17 | 0.412481 |
| FAM198B-AS1 | SIGLEC1 | 7.55E-17 | 0.412116 |
| AC020907.1 | MAG | 7.62E-17 | 0.412067 |
| AC023796.1 | CD22 | 7.71E-17 | 0.412008 |
| AC103770.1 | SIGLEC12 | 7.76E-17 | 0.41197 |
| AC138956.2 | SIGLEC1 | 7.83E-17 | 0.411923 |
| AP000695.2 | SIGLEC9 | 8.38E-17 | 0.411563 |
| LINC02126 | SIGLEC16 | 8.62E-17 | 0.411412 |
| ADPGK-AS1 | SIGLEC14 | 8.64E-17 | 0.4114 |
| NRIR | SIGLEC1 | 9.00E-17 | 0.411184 |
| AC007728.2 | SIGLEC5 | 9.42E-17 | 0.410938 |
| AC079921.1 | SIGLEC5 | 9.47E-17 | 0.410908 |
| AC073370.1 | SIGLEC7 | 1.00E-16 | 0.410615 |
| AL365361.1 | SIGLEC8 | 1.02E-16 | 0.410488 |
| AL133230.2 | SIGLEC1 | 1.05E-16 | 0.410349 |
| MIAT | SIGLEC1 | 1.08E-16 | 0.410211 |
| MUC12-AS1 | SIGLEC11 | 1.19E-16 | 0.409678 |
| AC025040.1 | SIGLEC1 | 1.20E-16 | 0.409651 |
| LINC02207 | SIGLEC9 | 1.29E-16 | 0.40925 |
| AC008840.1 | SIGLEC7 | 1.30E-16 | 0.409202 |
| AL050343.2 | SIGLEC1 | 1.33E-16 | 0.409108 |
| AC012181.2 | SIGLEC5 | 1.33E-16 | 0.409091 |
| AC008957.1 | SIGLEC1 | 1.43E-16 | 0.408705 |
| AC039056.2 | SIGLEC12 | 1.47E-16 | 0.408568 |
| TTTY7 | CD22 | 1.48E-16 | 0.408509 |
| GNA14-AS1 | SIGLEC16 | 1.51E-16 | 0.40839 |
| AC093278.2 | CD33 | 1.61E-16 | 0.408064 |
| LINC01943 | SIGLEC9 | 1.63E-16 | 0.407996 |
| LINC01146 | SIGLEC10 | 1.63E-16 | 0.407981 |
| AC007336.2 | SIGLEC9 | 1.64E-16 | 0.407952 |
| AP001476.2 | SIGLEC11 | 1.67E-16 | 0.407855 |
| AC005692.2 | SIGLEC16 | 1.70E-16 | 0.407757 |
| MACORIS | SIGLEC9 | 1.78E-16 | 0.407523 |
| AC005264.1 | SIGLEC1 | 1.78E-16 | 0.407513 |
| AL021707.5 | SIGLEC1 | 1.86E-16 | 0.407271 |
| MIMT1 | SIGLEC11 | 1.95E-16 | 0.407033 |
| AP001056.1 | SIGLEC1 | 1.97E-16 | 0.40697 |
| AOAH-IT1 | SIGLEC14 | 2.08E-16 | 0.406685 |
| LINC02285 | SIGLEC5 | 2.18E-16 | 0.406433 |
| AC020907.3 | MAG | 2.26E-16 | 0.406222 |
| AC087521.1 | SIGLEC11 | 2.34E-16 | 0.406039 |
| AL357054.4 | SIGLEC8 | 2.47E-16 | 0.405744 |
| AC008957.1 | SIGLEC8 | 2.57E-16 | 0.405529 |
| U62317.4 | SIGLEC14 | 2.71E-16 | 0.405238 |
| AC131097.3 | SIGLEC1 | 2.80E-16 | 0.405065 |
| AC084838.1 | SIGLEC16 | 2.82E-16 | 0.405016 |
| C1orf147 | SIGLEC9 | 2.83E-16 | 0.404999 |
| AC006369.1 | SIGLEC14 | 3.03E-16 | 0.404627 |
| AC004687.1 | SIGLEC7 | 3.16E-16 | 0.404412 |
| AC090409.1 | SIGLEC11 | 3.19E-16 | 0.404344 |
| LINC01934 | SIGLEC14 | 3.26E-16 | 0.404234 |
| AC100782.1 | SIGLEC6 | 3.27E-16 | 0.404221 |
| AC005999.1 | SIGLEC16 | 3.28E-16 | 0.404192 |
| WDFY3-AS2 | SIGLEC11 | 3.31E-16 | 0.404156 |
| LINC01638 | SIGLEC11 | 3.33E-16 | 0.404121 |
| AC079921.1 | SIGLEC9 | 3.44E-16 | 0.403944 |
| AC025887.1 | SIGLEC10 | 3.53E-16 | 0.403806 |
| AC090796.1 | CD33 | 3.53E-16 | 0.403803 |
| LINC01593 | SIGLEC6 | 3.54E-16 | 0.403787 |
| ADPGK-AS1 | CD33 | 3.58E-16 | 0.403727 |
| L3MBTL4-AS1 | SIGLEC8 | 3.70E-16 | 0.403537 |
| AC002511.1 | MAG | 3.72E-16 | 0.403519 |
| AC007608.3 | SIGLEC16 | 3.99E-16 | 0.403132 |
| AF111169.3 | SIGLEC1 | 4.06E-16 | 0.403034 |
| AC078850.1 | CD33 | 4.07E-16 | 0.403025 |
| MIR223HG | SIGLEC7 | 4.07E-16 | 0.403014 |
| LINC02568 | SIGLEC16 | 4.24E-16 | 0.4028 |
| AC004585.1 | CD33 | 4.35E-16 | 0.40266 |
| LINC02528 | CD33 | 4.54E-16 | 0.402415 |
| AC107204.1 | CD22 | 4.79E-16 | 0.402126 |
| LINC01891 | SIGLEC16 | 4.80E-16 | 0.402117 |
| KCNAB1-AS2 | SIGLEC11 | 4.93E-16 | 0.40197 |
| AL031658.1 | SIGLEC16 | 4.94E-16 | 0.401957 |
| AC138207.4 | SIGLEC9 | 4.95E-16 | 0.401939 |
| LINC00303 | MAG | 5.11E-16 | 0.401774 |
| AC073370.1 | SIGLEC9 | 5.13E-16 | 0.401751 |
| AC012181.2 | SIGLEC14 | 5.30E-16 | 0.401564 |
| FAM198B-AS1 | CD33 | 5.69E-16 | 0.401173 |
| LINC01150 | SIGLEC10 | 5.71E-16 | 0.401158 |
| AC100786.1 | SIGLEC11 | 5.81E-16 | 0.401062 |
| AC245128.3 | SIGLEC10 | 5.85E-16 | 0.401022 |
| AC092368.3 | SIGLEC9 | 5.96E-16 | 0.400919 |
| AC012404.2 | SIGLEC16 | 6.01E-16 | 0.400879 |
| AC008105.3 | SIGLEC9 | 6.16E-16 | 0.40074 |
| LINC02611 | SIGLEC9 | 6.40E-16 | 0.400529 |
| MACORIS | SIGLEC8 | 6.62E-16 | 0.40034 |
| AC006237.1 | MAG | 6.66E-16 | 0.400308 |
| AP003555.2 | SIGLEC10 | 6.68E-16 | 0.400292 |
| AC005392.1 | CD22 | 6.77E-16 | 0.400213 |
