# Supplementary material for: Relationships of SIGLEC family-related lncRNAs with clinical prognosis and tumor immune microenvironment in ovarian cancer
Source: Sci Rep. 2024 Mar 31;14:7593. doi: 10.1038/s41598-024-57946-7 (PMC10982283; doi:10.1038/s41598-024-57946-7)
Supplement: Supplementary file 4 — Supplementary Information 4. [file 41598_2024_57946_MOESM4_ESM.docx]

Supplementary table 3. Ninety-three SIGLEC family -related lncRNAs was obtained by univariate Cox regression analysis in TCGA-OV dataset.

| id | HR | HR.95L | HR.95H | pvalue |
| --- | --- | --- | --- | --- |
| AC039056.2 | 1.005699 | 1.002777 | 1.00863 | 0.000129 |
| AC008750.1 | 1.006008 | 1.002722 | 1.009306 | 0.000333 |
| AC245123.1 | 1.086547 | 1.037359 | 1.138067 | 0.000445 |
| PARM1-AS1 | 1.037786 | 1.016211 | 1.059819 | 0.00054 |
| AL160237.1 | 1.023669 | 1.010006 | 1.037517 | 0.000645 |
| AL137789.1 | 1.128647 | 1.049359 | 1.213927 | 0.001129 |
| AC073046.1 | 1.000811 | 1.000322 | 1.0013 | 0.00114 |
| LINC00955 | 1.036391 | 1.013868 | 1.059413 | 0.00143 |
| AL021878.2 | 1.004435 | 1.00168 | 1.007197 | 0.001587 |
| LINC02207 | 1.023157 | 1.008566 | 1.037959 | 0.001785 |
| AL590729.1 | 1.005954 | 1.002084 | 1.00984 | 0.002542 |
| AL121827.2 | 1.002174 | 1.000741 | 1.00361 | 0.002939 |
| AL355297.2 | 1.005596 | 1.00188 | 1.009327 | 0.003139 |
| AL356489.2 | 1.016469 | 1.00542 | 1.02764 | 0.003399 |
| LINC02798 | 1.01288 | 1.004037 | 1.021801 | 0.004228 |
| AC009292.1 | 1.006957 | 1.00217 | 1.011766 | 0.004348 |
| AC078788.1 | 0.981385 | 0.968568 | 0.994372 | 0.005087 |
| AC079584.1 | 1.041379 | 1.012053 | 1.071555 | 0.005403 |
| AC089999.4 | 1.003802 | 1.001097 | 1.006515 | 0.005844 |
| AL133279.1 | 1.02193 | 1.006232 | 1.037872 | 0.006023 |
| LINC01262 | 1.033818 | 1.009251 | 1.058983 | 0.006719 |
| AC097532.1 | 1.00518 | 1.001405 | 1.008968 | 0.00711 |
| AL008726.1 | 1.003086 | 1.000836 | 1.005341 | 0.007151 |
| LINC01250 | 1.007929 | 1.002142 | 1.013749 | 0.007178 |
| USP30-AS1 | 0.999254 | 0.998707 | 0.999802 | 0.007587 |
| AC093010.1 | 1.099742 | 1.025535 | 1.179318 | 0.007645 |
| AC026904.2 | 1.017346 | 1.00453 | 1.030325 | 0.007843 |
| AL008718.2 | 1.016336 | 1.004244 | 1.028574 | 0.007967 |
| AL357054.4 | 0.994693 | 0.990766 | 0.998635 | 0.008362 |
| AC007608.3 | 0.981983 | 0.968725 | 0.995423 | 0.008756 |
| LINC02035 | 1.001264 | 1.000302 | 1.002228 | 0.010039 |
| AC005330.1 | 1.003284 | 1.000777 | 1.005798 | 0.010223 |
| AC108058.1 | 1.003616 | 1.000849 | 1.00639 | 0.010392 |
| AC009145.1 | 1.017949 | 1.004141 | 1.031947 | 0.010678 |
| AL022238.3 | 1.005677 | 1.001281 | 1.010092 | 0.011309 |
| AL022318.1 | 1.005345 | 1.00114 | 1.009568 | 0.01267 |
| AC005253.1 | 1.002232 | 1.00047 | 1.003997 | 0.013014 |
| VSTM2A-OT1 | 1.537906 | 1.09043 | 2.169012 | 0.01415 |
| AC019080.1 | 1.007493 | 1.00148 | 1.013543 | 0.014527 |
| LINC01726 | 1.04036 | 1.007863 | 1.073905 | 0.014538 |
| AL355922.1 | 1.002777 | 1.000504 | 1.005056 | 0.016609 |
| AC138028.6 | 1.006297 | 1.001047 | 1.011575 | 0.018676 |
| MIR223HG | 1.001852 | 1.000303 | 1.003403 | 0.019127 |
| AL133230.2 | 1.011962 | 1.001923 | 1.022101 | 0.019406 |
| AC099560.1 | 1.006118 | 1.000975 | 1.011288 | 0.019667 |
| AL133371.2 | 1.002535 | 1.0004 | 1.004673 | 0.0199 |
| ATXN8OS | 1.013637 | 1.001998 | 1.025412 | 0.021524 |
| AP003774.2 | 1.000819 | 1.000117 | 1.001521 | 0.022129 |
| AL133467.1 | 0.992413 | 0.985953 | 0.998916 | 0.022285 |
| WARS2-IT1 | 1.016434 | 1.002306 | 1.030762 | 0.02246 |
| AC114811.2 | 1.026476 | 1.003477 | 1.050002 | 0.023813 |
| AL450998.3 | 1.002732 | 1.000327 | 1.005142 | 0.025938 |
| AC037198.2 | 1.001799 | 1.000207 | 1.003394 | 0.026789 |
| AC125494.1 | 1.012284 | 1.001318 | 1.023369 | 0.028016 |
| AC074131.1 | 0.976121 | 0.955154 | 0.997548 | 0.029144 |
| AC134312.3 | 1.002173 | 1.000206 | 1.004143 | 0.030321 |
| AC079949.1 | 1.000847 | 1.00008 | 1.001615 | 0.030523 |
| AC004522.4 | 1.007528 | 1.000702 | 1.0144 | 0.030606 |
| HCG22 | 1.003333 | 1.000305 | 1.00637 | 0.030947 |
| AC010731.3 | 1.022378 | 1.002029 | 1.043141 | 0.030963 |
| AC079921.1 | 1.007647 | 1.000696 | 1.014646 | 0.031008 |
| AC020658.5 | 1.004478 | 1.000371 | 1.008601 | 0.032582 |
| AC087286.2 | 1.004511 | 1.000367 | 1.008672 | 0.032859 |
| LINC00598 | 1.019892 | 1.001496 | 1.038626 | 0.033931 |
| AC079467.1 | 1.000994 | 1.000069 | 1.00192 | 0.03517 |
| LINC02040 | 1.063511 | 1.00429 | 1.126225 | 0.035171 |
| AL590867.1 | 1.013107 | 1.000879 | 1.025484 | 0.035569 |
| AL035670.1 | 0.941635 | 0.890269 | 0.995964 | 0.035618 |
| LINC01543 | 1.003219 | 1.000211 | 1.006236 | 0.035925 |
| AL031846.2 | 1.00901 | 1.000581 | 1.01751 | 0.036107 |
| AC007040.1 | 1.004719 | 1.000298 | 1.009159 | 0.036388 |
| AC245128.3 | 1.001609 | 1.000097 | 1.003122 | 0.037007 |
| LINC00211 | 1.057245 | 1.003317 | 1.11407 | 0.037165 |
| LINC01606 | 1.000471 | 1.000028 | 1.000915 | 0.037393 |
| FAM13A-AS1 | 1.007257 | 1.000415 | 1.014146 | 0.037604 |
| AC005692.1 | 0.847925 | 0.725763 | 0.990649 | 0.037679 |
| YEATS2-AS1 | 1.003029 | 1.000165 | 1.005902 | 0.038164 |
| AC087521.1 | 1.003643 | 1.000187 | 1.007111 | 0.03883 |
| AC008982.2 | 1.001105 | 1.000052 | 1.00216 | 0.039685 |
| AC092145.1 | 1.031684 | 1.001238 | 1.063057 | 0.041262 |
| FLNB-AS1 | 1.003908 | 1.000154 | 1.007676 | 0.041316 |
| TIMM23B-AGAP6 | 1.003521 | 1.000133 | 1.00692 | 0.041622 |
| AF111169.3 | 1.003976 | 1.000142 | 1.007826 | 0.042103 |
| AC124067.4 | 1.004109 | 1.000138 | 1.008096 | 0.042516 |
| AC022400.6 | 1.002499 | 1.000084 | 1.00492 | 0.042548 |
| AC027013.1 | 1.016157 | 1.000479 | 1.032082 | 0.043352 |
| AC112721.2 | 1.001496 | 1.000044 | 1.00295 | 0.043458 |
| AC090559.1 | 1.000786 | 1.000023 | 1.00155 | 0.043576 |
| AC011479.3 | 1.003854 | 1.000103 | 1.00762 | 0.044045 |
| PPM1F-AS1 | 1.01597 | 1.000414 | 1.031767 | 0.044157 |
| AC016910.2 | 1.036205 | 1.00083 | 1.07283 | 0.044775 |
| AC024337.2 | 1.008489 | 1.000157 | 1.016891 | 0.045825 |
| AC004946.2 | 1.05799 | 1.000183 | 1.119138 | 0.049257 |
