# Supplementary material for: Relationships of SIGLEC family-related lncRNAs with clinical prognosis and tumor immune microenvironment in ovarian cancer
Source: Sci Rep. 2024 Mar 31;14:7593. doi: 10.1038/s41598-024-57946-7 (PMC10982283; doi:10.1038/s41598-024-57946-7)
Supplement: Supplementary file 5 — Supplementary Information 5. [file 41598_2024_57946_MOESM5_ESM.docx]

Supplementary table 4: The regression coefficient of six SIGLEC family -related lncRNAs in risk model.

| id | coef |
| --- | --- |
| AC007608.3 | -0.01511 |
| AC008750.1 | 0.004533 |
| AC039056.2 | 0.004272 |
| AC078788.1 | -0.02664 |
| AL021878.2 | 0.00455 |
| AL133279.1 | 0.030881 |
